# Turner syndrome: French National Diagnosis and Care Protocol (NDCP; National Diagnosis and Care Protocol)

**DOI:** 10.1186/s13023-022-02423-5

**Published:** 2022-07-12

**Authors:** Elodie Fiot, Bertille Alauze, Bruno Donadille, Dinane Samara-Boustani, Muriel Houang, Gianpaolo De Filippo, Anne Bachelot, Clemence Delcour, Constance Beyler, Emilie Bois, Emmanuelle Bourrat, Emmanuel Bui Quoc, Nathalie Bourcigaux, Catherine Chaussain, Ariel Cohen, Martine Cohen-Solal, Sabrina Da Costa, Claire Dossier, Stephane Ederhy, Monique Elmaleh, Laurence Iserin, Hélène Lengliné, Armelle Poujol-Robert, Dominique Roulot, Jerome Viala, Frederique Albarel, Elise Bismuth, Valérie Bernard, Claire Bouvattier, Aude Brac, Patricia Bretones, Nathalie Chabbert-Buffet, Philippe Chanson, Regis Coutant, Marguerite de Warren, Béatrice Demaret, Lise Duranteau, Florence Eustache, Lydie Gautheret, Georges Gelwane, Claire Gourbesville, Mickaël Grynberg, Karinne Gueniche, Carina Jorgensen, Veronique Kerlan, Charlotte Lebrun, Christine Lefevre, Françoise Lorenzini, Sylvie Manouvrier, Catherine Pienkowski, Rachel Reynaud, Yves Reznik, Jean-Pierre Siffroi, Anne-Claude Tabet, Maithé Tauber, Vanessa Vautier, Igor Tauveron, Sebastien Wambre, Delphine Zenaty, Irène Netchine, Michel Polak, Philippe Touraine, Jean-Claude Carel, Sophie Christin-Maitre, Juliane Léger

**Affiliations:** 1 Pediatric Endocrinology-Diabetology Department, Reference Center for Rare Growth and Development Endocrine Diseases, INSERM NeuroDiderot, Assistance Publique-Hôpitaux de Paris, Université de Paris, Robert Debré University Hospital, 48 Bd Sérurier, 75019 Paris, France; 2 Department of Reproductive Endocrinology, Reference Center for Rare Growth and Development Endocrine Diseases, Assistance Publique-Hôpitaux de Paris, Sorbonne University, Saint Antoine Hospital, 75012 Paris, France; 3 Pediatric Endocrinology-Diabetology Department, Reference Center for Rare Growth and Development Endocrine Diseases, Assistance Publique-Hôpitaux de Paris, Université de Paris, Necker Enfants Malades University Hospital, 75015 Paris, France; 4 Explorations Fonctionnelles Endocriniennes, Reference Center for Rare Growth and Development Endocrine Diseases, Assistance Publique-Hôpitaux de Paris, Sorbonne University, Armand-Trousseau Hospital, 75012 Paris, France; 5 Endocrinology and Reproductive Medicine Department, Reference Center for Rare Growth and Development Endocrine Diseases, Assistance Publique-Hôpitaux de Paris, Sorbonne University, Pitié Salpétrière University Hospital, 75013 Paris, France; 6 Department of Obstetrics and Gynecology, Assistance Publique-Hôpitaux de Paris, Université de Paris, Robert Debré University Hospital, 75019 Paris, France; 7 Cardiopaediatric Unit, Assistance Publique-Hôpitaux de Paris, Université de Paris, Robert Debré University Hospital, 75019 Paris, France; 8 Pediatric Otorhinolaryngology Department, Assistance Publique-Hôpitaux de Paris, Université de Paris, Robert Debré University Hospital, 75019 Paris, France; 9 Dermatology Unit, Assistance Publique-Hôpitaux de Paris, Université de Paris, Robert Debré University Hospital, 75019 Paris, France; 10 Ophthalmology Department, Assistance Publique-Hôpitaux de Paris, Université de Paris, Robert Debré University Hospital, 75019 Paris, France; 11 Odontology Department, Assistance Publique-Hôpitaux de Paris, University Hospitals Charles Foix, PNVS, and Henri Mondor, 94000 Créteil, France; 12 Department of Cardiology, GRC n°27, GRECO, AP-HP, Assistance Publique-Hôpitaux de Paris, Sorbonne University, Saint Antoine Hospital, 75012 Paris, France; 13 Department of Rheumatology, Assistance Publique-Hôpitaux de Paris, Université de Paris, Lariboisière Hospital, 75010 Paris, France; 14 Reference Center for Rare Gynecological Pathologies, Pediatric Endocrinology Department, Assistance Publique-Hôpitaux de Paris, Université de Paris, Necker Enfants Malades University Hospital, 75015 Paris, France; 15 Department of Paediatric Nephrology, Assistance Publique-Hôpitaux de Paris, Université de Paris, Robert Debré University Hospital, 75019 Paris, France; 16 Department of Radiology, Assistance Publique-Hôpitaux de Paris, Université de Paris, Robert Debré University Hospital, 75019 Paris, France; 17 Adult Congenital Heart Disease Unit, Cardiology Department, Assistance Publique-Hôpitaux de Paris, Université de Paris, Georges Pompidou University Hospital, 75015 Paris, France; 18 Department of Pediatric Gastroenterology and Nutrition, Assistance Publique-Hôpitaux de Paris, Université de Paris, Robert Debré University Hospital, 75019 Paris, France; 19 Hepatology Department, Assistance Publique-Hôpitaux de Paris, Sorbonne University, Saint Antoine Hospital, 75012 Paris, France; 20 Hepatology Department, Assistance Publique-Hopitaux de Paris, Université Sorbonne Paris Nord, Avicenne Hospital, 93009 Bobigny, France; 21 Department of Endocrinology, Assistance Publique-Hôpitaux de Marseille. Hospital La Conception, 13005 Marseille, France; 22 Department of Pediatric Endocrinology and Diabetology, Competence Center for Rare Diseases of Insulin Secretion and Insulin Sensitivity, Assistance Publique-Hôpitaux de Paris, Université de Paris, Robert Debré University Hospital, 75019 Paris, France; 23 CHU Pellegrin, Department of Gynecological Surgery, Medical Gynecology and Reproductive Medicine, Centre Aliénor d’aquitaine, Bordeaux University Hospitals, 33000 Bordeaux, France; 24 Paediatric Endocrinology Department, Reference Center for Rare Genital Development Disorders, Assistance Publique-Hôpitaux de Paris, Kremlin-Bicêtre University Hospital, Paris-Sud University, 94270 Le Kremlin-Bicêtre, France; 25 Department of Endocrinology Pediatric and Adult, Reference Center for Rare Genital Development Disorders, Lyon Hospices Civils, Est Hospital Group, 69677 Bron, France; 26 Gynecology-Obstetrics and Reproductive Medicine Department, Assistance Publique-Hôpitaux de Paris, Tenon University Hospital, 75020 Paris, France; 27 Department of Endocrinology and Reproductive Diseases, Assistance Publique-Hôpitaux de Paris, Kremlin-Bicêtre University Hospital, Paris-Sud University, 94270 Le Kremlin-Bicêtre, France; 28 Department of Pediatric Endocrinology and Diabetology and Reference Center for Rare Diseases of Thyroid and Hormone Receptivity, Angers University Hospital, 49100 Angers, France; 29 AGAT, French Turner Syndrome Association (AGAT; Association Des Groupes Amitié Turner), 75011 Paris, France; 30 Grandir Association (French Growth Disorders Association), 92600 Asnières-sur-Seine, France; 31 Adolescent and Young Adult Gynecology Unit, Reference Center for Rare Genital Development Disorders, Assistance Publique-Hôpitaux de Paris, Kremlin-Bicêtre University Hospital, Paris-Sud University, 94270 Le Kremlin-Bicêtre, France; 32 Reproductive Biology Department, Assistance Publique-Hôpitaux de Paris, Jean Verdier University Hospital, 93140 Bondy, France; 33 Department of Endocrinology and Metabolic Diseases, Caen University Hospital, 14000 Caen, France; 34 Department of Reproductive Medicine and Fertility Preservation, Assistance Publique-Hôpitaux de Paris, Antoine Béclère University Hospital, 92140 Clamart, France; 35 Endocrinology and Metabolism Department, Assistance Publique-Hôpitaux de Paris, Sorbonne University, Saint Antoine Hospital, 75012 Paris, France; 36 Endocrinology and Metabolism Department, Brest University Hospital Centre, 29200 Brest, France; 37 Pediatric Endocrinology, Lille University Jeanne de Flandre Hospital, 59000 Lille, France; 38 Department of Endocrinology, Toulouse University Paule Viguier Hospital, 31300 Toulouse, France; 39 Clinical Genetics Department, DEV GEN Genital Development Reference Center, Lille University Jeanne de Flandre Hospital, 59000 Lille, France; 40 Genetics and Medical Gynecology Department, Reference Center for Rare Gynecological Pathologies, Toulouse University Hospitals - Hôpital Des Enfants, Pediatrics - Endocrinology, 31059 Toulouse, France; 41 Department of Multidisciplinary Pediatrics, Reference Center for Pituitary Rare Diseases Aix Marseille University, Assistance Publique-Hôpitaux de Marseille, Hôpital de La Timone Enfants, 13005 Marseille, France; 42 Genetics and Embryology Department, Sorbonne Université; INSERM UMRS-933, Assistance Publique-Hôpitaux de Paris, Hôpital d’Enfants Armand-Trousseau, Paris, France; 43 Genetics Department, Assistance Publique-Hôpitaux de Paris, Université de Paris, Robert Debré University Hospital, 75019 Paris, France; 44 Genetics and Medical Gynecology Department, Toulouse University Hospital - Hôpital Des Enfants, Pediatrics - Endocrinology, 31059 Toulouse, France; 45 Pediatric Diabetology Department, Bordeaux University Hospitals, 33000 Bordeaux, France; 46 Clermont-Ferrand University Hospital, Endocrinology Department, Clermont Auvergne University, 63000 Clermont-Ferrand, France; 47 French Turner Syndrome Association (Turner Et Vous Association), 59155 Faches-Thumesnil, France

**Keywords:** Turner’s syndrome, Childhood, Adulthood, Diagnosis, Recommendation, Management

## Abstract

Turner syndrome (TS; ORPHA 881) is a rare condition in which all or part of one X chromosome is absent from some or all cells. It affects approximately one in every 1/2500 liveborn girls. The most frequently observed karyotypes are 45,X (40–50%) and the 45,X/46,XX mosaic karyotype (15–25%). Karyotypes with an X isochromosome (45,X/46,isoXq or 45,X/46,isoXp), a Y chromosome, X ring chromosome or deletions of the X chromosome are less frequent. The objective of the French National Diagnosis and Care Protocol (PNDS; *Protocole National de Diagnostic et de Soins*) is to provide health professionals with information about the optimal management and care for patients, based on a critical literature review and multidisciplinary expert consensus. The PNDS, written by members of the French National Reference Center for Rare Growth and Developmental Endocrine disorders, is available from the French Health Authority website. Turner Syndrome is associated with several phenotypic conditions and a higher risk of comorbidity. The most frequently reported features are growth retardation with short adult stature and gonadal dysgenesis. TS may be associated with various congenital (heart and kidney) or acquired diseases (autoimmune thyroid disease, celiac disease, hearing loss, overweight/obesity, glucose intolerance/type 2 diabetes, dyslipidemia, cardiovascular complications and liver dysfunction). Most of the clinical traits of TS are due to the haploinsufficiency of various genes on the X chromosome, particularly those in the pseudoautosomal regions (PAR 1 and PAR 2), which normally escape the physiological process of X inactivation, although other regions may also be implicated. The management of patients with TS requires collaboration between several healthcare providers. The attending physician, in collaboration with the national care network, will ensure that the patient receives optimal care through regular follow-up and screening. The various elements of this PNDS are designed to provide such support.

## Summary for general practitioners

Turner syndrome (TS) is a rare genetic disorder in which all or part of one X chromosome is absent: karyotype 45,X, mosaicism 45,X/46,XX, with possible variations of the mosaicism, presence of the Y chromosome, structural abnormalities of the X chromosome, such as X isochromosome (45,X/46,isoXq or 45,X/46,isoXp), ring X chromosome or deletion of the X chromosome. TS is almost always associated with short stature and ovarian insufficiency. Several other features have been described in some, but not all patients: morphological characteristics of various intensities, congenital malformations, and a high risk of acquired comorbid conditions (“Appendix 1”). Cognitive performance is generally satisfactory, although some patients have difficulty with certain kinds of learning, and some very specific abnormalities of the X chromosome (ring) may be accompanied by intellectual disability.

It is important for the treating physician:

- To consider this diagnosis, for a girl, as soon as possible after the following have been observed:
  - *Female neonate:* lymphedema of the extremities, aortic coarctation/bicuspid aortic valve;
  - *Female infant or child:* short stature (height ≤  − 2 SD or height <  − 1.5 SD relative to the target parental height) regardless of growth rate, or low height velocity, with or without a clinical phenotype suggestive of TS (“Appendix 1”), medical history of aortic coarctation/bicuspid aortic valve;
  - *Adolescent*: short stature (≤ − 2 SD) with or without a suggestive clinical phenotype (“Appendix 1”), delayed onset of puberty with no breast development after the age of 13 years, pubertal arrest, primary or secondary amenorrhea with high serum gonadotropin concentrations;
  - *Adult*: short stature, suggestive clinical phenotype, primary or secondary amenorrhea with high serum gonadotropin concentrations, or infertility.
- To refer the patient for consultation with a hospital specialist (a pediatric endocrinologist with specialist experience with TS, an adult endocrinologist, or a medical gynecologist) for definitive diagnosis, information of the patient and specialized multidisciplinary management (short stature, premature ovarian insufficiency, cardiac or renal malformations, screening for possible associated conditions: autoimmune, metabolic, ENT, hepatic, gastroenterological, etc.) (“Appendix 2”).
- To confirm the diagnosis by blood karyotyping or a FISH test on the sex chromosomes.
  The diagnosis should be announced as part of the overall management process.
- To ensure that the patient is undergoes multidisciplinary management in a hospital environment, with coordination by a reference or expert center (multidisciplinary center) in conjunction with the Rare Disease network (ENDO-ERN network for European centers). If the patient is a child, management should involve the pediatrician and/or the general practitioner. For adult patients, management should involve a local endocrinologist or medical gynecologist working together with the general practitioner.
- Therapeutic management at the hospital:
  - Short stature: treatment with recombinant growth hormone (rhGH) should be started early, when height is ≤ -− 2 SD or when a significant decrease in height velocity is detected;
  - Premature ovarian insufficiency: induction of puberty with estrogen at a normal pubertal age (except for forms with a spontaneous pubertal onset, which may nevertheless require secondary treatment if puberty does not progress). Hormonal replacement therapy (HRT) appropriate for the adult period and adapted for risk factors (except in the presence of very rare contraindications) should be continued at least until the physiological age of menopause (50 years). Subsequent reassessment should take place with the doctor on a case-by-case basis, to ensure the prevention of cardiovascular and bone risks. About 7% of patients, on average, manage to fall pregnant naturally, particularly those with mosaic 45,X/46,XX karyotypes. Medically assisted reproduction by in vitro fertilization of a donated egg may be successful, provided that the patient’s cardiovascular and hepatic assessments are compatible with pregnancy. In cases of spontaneous puberty with persistent natural cycles, fertility preservation should be discussed.
  - Screening and appropriate treatment of any associated illnesses (hearing loss, arterial hypertension (HBP), dysthyroidism, diabetes, dyslipidemia, etc.) and possible difficulties at school or with socioprofessional integration.
- Monitoring by the pediatrician and/or general practitioner (“Appendix 3”):
  - Prescribed treatments (adverse effects, compliance);
  - Regular BP checks;
  - Screening for associated illnesses (functional signs);
  - Lifestyle, psychological state, management of cardiovascular risk factors, prevention and management of overweight/obesity;
  - Antibiotic prophylaxis to prevent endocarditis, if necessary.
- Appropriate psychological support (psychologist, psychiatrist) with personalized assistance, if necessary:
  - Pediatric period: speech therapy, learning support at school, help with psychomotor skills, etc.
  - Adults: help with social relationships, professional integration, etc.
- Doctors should ensure that patients benefit through all the available help through ALD status [*affections de longue durée*, benefits and accommodation available for people with long-term conditions] and the MDPH [*Maison départementale des personnes handicapées*, the local authority for disabled people).
- Information about all the available patient associations should be provided.
- Follow-up should be regular (every 6 to 12 months), and should take place at the hospital, for patients of all ages. Local follow-up is possible for adults, provided it is performed in collaboration with a reference or multidisciplinary center, together with the Rare Endocrine Diseases network.
  - If the patient is followed by a local specialist during adulthood (endocrinologist, gynecologist experienced in the management of TS), an assessment (clinical and paraclinical examinations) should be performed in a hospital environment at a reference or expert center at least every five years, and at shorter intervals if the patient has associated conditions, particularly if they are cardiovascular in nature (“Appendix 3”).
- Any concomitant diseases should be treated by the general practitioner (or pediatrician), if necessary in partnership with a doctor from the reference center or expert center;
  - Effective antibiotic treatment should be administered in cases of acute otitis media, with systematic otoscopic follow-up during treatment, due to the high risk of deafness in these patients;
  - Antibiotic prophylaxis should be administered in the event of bicuspid aortic valve or other valvulopathy (dental care, surgery, etc.);
  - Patients should be screened and, if necessary, treated for urinary tract infections, if they have a urinary tract malformation.
  - Education should be given about the drugs likely to prolong the QT interval (list provided by the cardiologist).

## Introduction

Turner syndrome (TS) is a rare genetic disorder in which all or part of one X chromosome is absent. It affects one in every 2500 female newborns. It is almost always associated with short stature and ovarian insufficiency. Other features have been described, but are not present in all patients: morphological characteristics of various intensities, congenital malformations, and a high risk of acquired comorbid conditions (“Appendix 1”). Cognitive performance is generally satisfactory, although some patients have difficulty with certain kinds of learning, and some very specific abnormalities of the X chromosome (ring) may be accompanied by intellectual disability.

Patients are generally of normal intelligence, but some have a particular neuropsychological profile with a high degree of anxiety, which may require appropriate management (difficulties with mathematics, difficulties with visuospatial orientation, attention deficits, difficulties with fine motor skills, memory problems, low self-esteem, etc.).

The diagnosis is established after karyotyping or a FISH analysis on the sex chromosomes (on blood, tissues, buccal cells, or amniotic fluid), which reveals 45,X monosomy in approximately 40–50% of cases. The other forms consist essentially of mosaics (45,X/46,XX, possible variability of mosaicism), the presence of the Y chromosome, or structural abnormalities of the X chromosome, such as X isochromosome (45,X/46,isoXq or 45,X/46,isoXp), ring X chromosome or X chromosome deletion. In patients with mosaicism, at least 5% of the cells must be 45,X cells for the diagnosis to be established. Some patients may have 45,X/46,XY mosaic forms with the presence of Y chromosome material. In cases of antenatal diagnosis, karyotyping should be performed postnatally.

Treatment with rhGH during the pediatric period is recommended to increase the predicted height and final adult height of these patients. Puberty induction is often required, and is achieved with low doses of estrogens followed by hormone replacement therapy combining estrogen and progesterone/progestogen for premature ovarian insufficiency.

Turner Syndrome is associated with an increase in the risk of congenital malformations and comorbid conditions (cardiac and/or vascular, renal, bone, ENT, metabolic, endocrine, autoimmune, hepatic, gastroenterological, stomatological [oral medicine], psychological, etc.).

The treatment of male subjects with 45,X/46,XY karyotypes will not be discussed separately in this document. In such patients, management and strategies for detecting comorbid conditions are similar to those for patients with Turner Syndrome.

## Objectives of the national diagnosis and care protocol

The objective of this national diagnosis and care protocol (NDCP) is to describe current optimal diagnostic and therapeutic management and the integrated care pathway for Turner Syndrome (TS) in pediatric and adult patients.

This NDCP was drawn up according to the "Method for drafting a national protocol for the diagnosis and care of rare diseases" published by the French National Health Authority (HAS) in 2012 (methodology guide available on the HAS website: www.has-sante.fr). It is based on several original international publications, journals and international consensus conferences. In some cases, in the absence of published data, the editors propose consensus approaches based on the experience of group members, supplemented with expert advice. This NDCP will be updated as and when new data are published and validated. This NDCP does not claim to include all possible management approaches and cannot replace the individual responsibility of specialist physicians for their patients.

The specific aims of this NDCP include:

- Improving antenatal management and the announcement of the diagnosis;
- Screening for and managing possible associated comorbid conditions, to decrease morbidity and mortality;
- Optimizing growth and puberty;
- Ensuring continuity of care and facilitating the transition from pediatric to adult healthcare;
- Providing guidelines for the necessary follow-up during adulthood;
- Improving quality of life for both pediatric and adult patients.

- Standardizing practices

This NDCP can be used as a reference by attending physicians working with other medical specialists. It will be periodically updated, based on new validated data. Certain recommendations, provided by French healthcare providers, may not be feasible in certain countries, mostly outside Europe and North America, with limited possibilities for medical evaluation and treatment. The recommendations should, therefore, be interpreted in the context of the resources available.

An additional document comprising the identified bibliographic sources (scientific support) is available from the HAS website at (http://www.has-sante.fr) and from the website of the *Centre de Référence des Maladies Endocriniennes Rares de la Croissance et du Développement*-CRMERCD- (http://crmerc.aphp.fr). (in French).

## Initial diagnosis and evaluation

### Objectives

- To favor the earliest possible diagnosis of Turner syndrome;
- To confirm the diagnosis of Turner syndrome;
- To provide information concerning the need for postnatal karyotyping even if antenatal karyotyping has already been performed, and about screening for Y material, given the associated risk of gonadoblastoma;
- To inform doctors that, in cases of structural abnormalities of the X chromosome, parental karyotyping should systematically be performed, even though most of these abnormalities occur de novo. Genetic counseling may be necessary;
- To promote screening for congenital malformations (cardiac/vascular and renal) and/or associated comorbidities;
- To provide information about the risk of short stature and premature ovarian insufficiency and the corresponding treatments. To provide information about the possibility of medically assisted procreation (MAP) with egg donation. Spontaneous pregnancies may occur but are rare (7%). To provide information about the risks of pregnancy and the need for special monitoring for TS. In cases of spontaneous puberty and consistent natural cycles, fertility preservation should be discussed.
- To provide information about the risk of autoimmune diseases, acquired cardiovascular diseases, ENT involvement, subsequent metabolic risk (HBP, dyslipidemia, overweight, carbohydrate intolerance, diabetes, etc.) and hepatic and gastroenterological diseases;
- To provide information about possible difficulties with certain kinds of learning, which can be managed, although cognitive performance is generally satisfactory;
- To identify the key educational skills to be acquired;
- To present the modes of periodic monitoring of the disease.
- To suggest systematic psychological evaluation and management;
- To provide contact information for patient organizations

### Professionals involved (and coordination)

The diagnosis, initial assessment and management of the patient require multidisciplinary cooperation between various healthcare professionals, coordinated by the hospital specialist (pediatric endocrinologist, pediatrician experienced in TS, endocrinologist or gynecologist), at a reference or expert center or locally but in collaboration with an expert center.

- Referral specialist: pediatric endocrinologist, pediatrician experienced in TS, endocrinologist, gynecologist.
- Other specialist physicians: cardiologist, ENT specialist, gynecologist, clinical geneticist, cytogeneticist, nephrologist, orthopedist, rheumatologist, gastroenterologist, hepatologist, stomatologist/oral medicine specialist, ophthalmologist, dermatologist, psychiatrist or child psychiatrist, reproductive medicine doctor, plastic surgeon.
- Other health professionals: psychologist, psychomotor therapist and/or speech therapist, social worker (if necessary), educational therapy nurse, social worker.

This multidisciplinary management is necessary to provide comprehensive patient care.

### Initial evaluation

The initial evaluation will make it possible:

- To announce a diagnosis of TS.
- To perform a complete clinical examination;
- To schedule the paraclinical examinations necessary to detect any possible malformations and/or associated disorders.

#### Diagnosis circumstances/criteria

Karyotyping or FISH analysis on sex chromosomes to screen for TS is indicated in the following situations:

- *During the antenatal period*: suspicious signs on ultrasound (“Appendix 1”).
- *In female newborns*: lymphedema of the extremities, thick neck, abnormalities of the left heart (aortic coarctation, bicuspid aorta, hypoplasia of the left heart, etc.), clinical phenotype suggestive of TS (“Appendix 1”).
- *Female infant or child***:** short stature (height ≤ − 2 SD or height ≤ − 2 SD relative to parental target height) regardless of growth rate, or decrease in height velocity, medical history of aortic coarctation; with or without a clinical phenotype suggestive of TS (“Appendix 1”).
- *Adolescent*: short stature (≤ -− 2 SD) with or without a suggestive clinical phenotype, delayed onset of puberty with no breast development after the age of 13 years, no progression of puberty, primary or secondary amenorrhea with high serum gonadotropin concentrations;
- *Adult*: short stature, suggestive clinical phenotype, primary or secondary amenorrhea with high serum gonadotropin concentrations, recurrent miscarriages and/or infertility.
- The definitive diagnosis is established by FISH analysis on the sex chromosomes or karyotyping (on blood lymphocytes, amniotic fluid, buccal cells, etc.). The analysis of at least 20 cells was recommended by the last consensus conference, published in 2017 [1]. In certain cases, screening may be performed for possible mosaicism on a larger number of cell nuclei, on 100 or 200 cells (routinely performed with the FISH technique) [2].

#### Clinical evaluation

**The initial diagnosis investigations are described in “Appendix **2”

**Childhood**

- Evaluate weight, height, puberty stage, body mass index (BMI), blood pressure (BP) after 10 min of rest, preferably in both arms, or at least in the right arm (especially if there is a history of aortic coarctation), cardiac auscultation, monitoring of nevi. Peripheral pulses and examination of hips in infancy.
- Look for characteristic morphological features (“Appendix 1”).
- Evaluate possible scoliosis, kyphosis.
- Screen for hearing loss.
- Analyze growth (standard and Turner growth curves).
- Provide information, appropriate for the child's age, on the following:
  - The risks of short stature and premature ovarian insufficiency with delayed puberty, amenorrhea and infertility, and the corresponding treatments;
  - The possible risk of cardiac or renal malformations, ENT involvement, acquired cardiovascular diseases (HBP and dilated aorta), autoimmune diseases, subsequent metabolic abnormalities (dyslipidemia, overweight, carbohydrate intolerance, diabetes, etc.);
  - The possible existence of difficulties with certain kinds of learning, although cognitive performance is generally satisfactory.
- Assess academic level and adaptation to schooling.

**Adulthood**

- Clinical examination: weight, height, BMI, BP after 10 min of rest, preferably in both arms, and at least in the right arm (especially if there is a history of aortic coarctation), cardiac auscultation, characteristic morphological features, monitoring of nevi (“Appendix 1”), and breast development. During the medical interview, check for functional signs: cardiac (dyspnea, angina, syncope), digestive (diarrhea, blood in the stools), menstrual cycle disorders or bleeding related to HRT, estrogen deficiency, dysthyroidism, hearing difficulties.
- Provide information about premature ovarian insufficiency, its consequences for infertility, and its treatment. Provide information about the importance of hormone replacement therapy at least until the physiological age of menopause (about 50 years of age), with subsequent re-assessment of this need with the doctor on an individual basis, to prevent skin, cardiovascular and bone risks and to optimize the patient’s sexuality.
- Discuss the possibilities of pregnancy through MAP (in vitro fertilization with egg donation), explaining the possible risks in the event of pregnancy, including the risk of cardiovascular disease and the contraindication of pregnancy in certain cases. Discuss fertility preservation (egg freezing) in the event of spontaneous menstrual cycles.
- Provide information about current and future management and screening for potential associated comorbidities: acquired cardiovascular diseases (HBP -high blood pressure-, dilated aorta), ENT diseases, autoimmune diseases (thyroid, celiac disease, etc.), later-life metabolic risk (dyslipidemia, overweight, carbohydrate intolerance, diabetes, etc.), stressing the importance of regular long-term multidisciplinary follow-up.
- Evaluate the patient’s overall experience of TS, quality of life, self-esteem, sexuality and socioprofessional integration.

#### Paraclinical evaluation

**The paraclinical evaluations** to be performed early in patient management are described in “Appendix 2”. These examinations should be adapted according to the patient’s age and diagnostic circumstances. They are performed to screen for possible cardiac or renal malformations and for other potentially associated diseases.

#### Antenatal diagnosis

The parents should be provided with information about TS. There is no correlation between the antenatal karyotype and clinical phenotype or the risks of subsequent acquired disorders. The possibility of a less severe, or even normal, phenotype in cases of mosaicism or minor karyotype abnormality should be mentioned, and it should be explained that most patients with TS have normal intelligence. However, the existence, in some cases, of antenatal ultrasound signs of a more severe condition (cervical cystic hygroma, anasarca, severe cardiopathy) should also be taken into account. For formal announcement of the diagnosis, the following should be done promptly:

- A consultation should be organized between the parents (both if possible) and a clinical geneticist or cytogeneticist, with a pediatric endocrinologist in attendance. Ideally, both parents should be present, and accompanied by a psychologist.
- The parents should be informed of the risk of short stature, ovarian insufficiency and its consequences in terms of infertility, and about the treatments for these conditions.
- Information should be provided concerning the risks of characteristic morphological features, ENT involvement, cardiac (bicuspid aorta) and vascular diseases (aortic coarctation and dilation of the ascending aorta), autoimmune diseases, hepatic abnormalities and later-life metabolic risk (hypertension, dyslipidemia, overweight, carbohydrate intolerance, diabetes, etc.) requiring long-term multidisciplinary monitoring.
- It should be explained that most patients lead productive happy lives and are of normal intelligence. However, the possibility of a particular neurocognitive profile, problems with social skills, and, in some cases, moderate learning difficulties (not systematic, and often associated with particular cytogenetic abnormalities, such as ring X chromosomes) should be mentionned.
- A fetal ultrasound scan should be performed to screen for any associated cardiac or renal malformations (not present in all patients).
- Psychological therapy should be proposed for the parents.
- The parents should be advised to contact patient associations.
- Information about the need for postnatal karyotyping should be provided.
- Due to the potential severity of certain forms, the diagnosis may lead to requests, by the mother or the couple, to terminate the pregnancy for medical reasons. Such requests should be examined by a prenatal diagnostic multidisciplinary center.

#### Neonatal management

Neonatal management can be organized by the maternity unit pediatrician in partnership with a clinical geneticist and/or a pediatric endocrinologist.

- Detailed clinical examination, with screening for characteristic morphological features.
- Screening for aortic coarctation (peripheral pulses, BP), examination of the hips.
- FISH analysis on sex chromosomes or karyotyping. Screening for Y chromosome material (FISH or PCR): molecular analysis is indicated to detect Y-chromosome sequences in TS patients, regardless of their karyotype, but there is no need to check for Y chromosome material in patients with structural abnormalities, such as isoXq or Xp, if there is no mosaicism. In cases of ring X chromosomes, microarray analysis to check for presence of the *XIST* gene.
- Consultation with a cardiologist, in a pediatric setting, with cardiac ultrasound (screening for malformations) and, possibly, electrocardiogram (EKG) (QT measurement) examinations.
- Renal ultrasound examination to screen for malformations.
- Consultation with a pediatric endocrinologist at about the age of 6 to 12 months, or earlier if requested by the parents.
- Gonadotrophin determination.
- Plasma creatinine determination, if renal abnormalities are detected.

#### Childhood management

**Announcement of the diagnosis:**

- Provide information about the risk of short stature and premature ovarian insufficiency and infertility, and the corresponding treatments: rhGH, hormone remplacement therapy (HRT), egg donation in adulthood; post-puberty egg freezing in rare cases of spontaneous puberty, for which the long-term outcomes remain uncertain.
- Provide information about the possible risks of cardiac (bicuspid aorta) and vascular (aortic coarctation and dilation of the ascending aorta) diseases and their consequences for TS management, the risk of ENT involvement, autoimmune diseases, and later-life metabolic risks (hypertension, dyslipidemia, overweight, carbohydrate intolerance, diabetes, etc.).
- Provide information about the possibility of a particular neurocognitive profile, sometimes with moderate learning difficulties, requiring specific management and often associated with particular cytogenetic abnormalities, such as ring X chromosomes. It should be pointed out that most patients with TS are of normal intelligence.

**Clinical and paraclinical evaluation (“**Appendix 2”)**:**

- Analysis of the standard and Turner syndrome growth curves.
- Clinical examination (weight, height, BMI, puberty stage, screening for a heart murmur, bruits, BP after 10 min of rest, preferably in both arms, and at least in the right arm (especially if there is a history of aortic coarctation), peripheral pulses, screening for morphological features, skin signs, examination of the hips in infants); evaluation of possible scoliosis, kyphosis.
- Take blood samples to check for possible associated comorbidities:
  - FSH, LH, AMH, estradiol determinations from the age of 10 years;
  - Detection of antibodies against thyroid peroxidase (anti-TPO Ab), TSH, ± FT4, from the age of four years, or from diagnosis;
  - HbA1c ± glucose determinations, starting from the age of 10 years or before the start of GH treatment;
  - OGTT (oral glucose tolerance test) if a moderate increase in HbA1c and/or blood sugar levels is observed; fasting insulin in blood (HOMA) if the patient is overweight;
  - Triglyceride, total cholesterol, HDL and LDL levels in a fasting state at the end of puberty, and then between the ages of 17 and 21 years, or before if there are risk factors (overweight, family history of dyslipidemia);
  - AST, ALT, gamma GT, ALP determinations, from the age of 6 years;
  - Antitransglutaminase (IgA) Ab determinations, from the age of two to three years, or earlier in cases of insufficient weight gain and/or abdominal pain;
  - Blood creatinine determination and screening for microalbumin in the urine in cases of renal abnormality or high BP;
  - CBC (complete blood count), checking for the presence of anemia;
  - 25(OH)D [25-hydroxy vitamin D] determination.
- Screening for Y chromosome material (FISH or PCR), if not done previously. Molecular analysis is indicated to detect Y-chromosome sequences in TS patients, regardless of their karyotype, but there is no need to check for Y chromosome material in patients with structural abnormalities, such as isoXq or Xp, in the absence of mosaicism.
- Bone age evaluation (X rays of the left wrist and hand) before rhGH treatment.
- Pelvic ultrasound (or even MRI) scans if Y chromosome material is found, to check for gonadoblastoma.
- Consultation with a cardiologist in a pediatric setting, with cardiac ultrasound (screening for malformations, use of Z scores to assess aortic dimensions) and electrocardiogram (EKG) (QT measurement) examinations; a list of contraindicated drugs should be provided by the cardiologists if a long QT is found.
- Renal ultrasound scan to screen for possible malformations.
- ENT consultation in a pediatric setting, with hearing tests performed with an age-appropriate technique.
- Ophthalmology consultation, best performed at the age of 12–15 months if diagnosis occurs early enough, to screen for associated abnormalities responsible for amblyopia (strabismus, refractive anomalies). A regular consultation, as for the general population, should take place between the ages of three and four years, and at diagnosis, if diagnosis occurs later. Ophthalmological examinations should be performed, at any age, in cases of suspicious visual signs or characteristic clinical signs.
- Annual or biannual consultations should take place with a specialist dentist in the early pediatric period (oral hygiene, fluoride, preventive sealing of pits and fissures, etc.) and throughout the period of growth. If necessary, panoramic X rays can be performed, from the age of seven years. Patients should consult an orthodontist, if necessary.
- Evaluation of dietary intake of calcium and vitamin D.
- Neuropsychological assessment, possibly supplemented with psychometric tests in the event of learning difficulties, from the age of four or five years, depending on clinical profile, or earlier in cases of suspicious signs, with appropriate management, if necessary.
- Psychological consultation should be systematically suggested, with management, if necessary;
- A visit to the social worker should be suggested, and, if necessary, the MDPH [*Maison Départementale pour les Personnes Handicapées*, the local authority for disabled people].
- The parents should be advised to contact patient organizations.

#### Adult patient management

- Clinical examination (height, weight, and, if possible, waist size and waist-to-height ratio (WHtR), BMI, screening for a heart murmur, BP after 10 min of rest, preferably in both arms, at least in the right arm (especially if there is a history of aortic coarctation), screening for morphological features and functional signs).
- Provide information about the impact of TS on height deficit, for which treatment with growth hormone cannot be offered after fusion of the growing cartilage.
- Provide information about the risk of premature ovarian insufficiency, and its consequences in terms of infertility, its treatment and the importance of long-term replacement therapy, at least until the physiological age of menopause (50 years). Suggest fertility preservation in the absence of premature ovarian insufficiency, even if its long-term outcomes remain unclear. Provide information about the possibility of pregnancy by egg donation in the event of premature ovarian insufficiency, specifying the need for regular monitoring during pregnancy because of the possible risks incurred, including risks of cardiovascular and/or liver disease. There may be absolute contraindications for pregnancy in certain cases.
- Provide information about the possible risk (not present in all patients) of acquired cardiovascular diseases, ENT involvement, autoimmune diseases, metabolic risk (hypertension, dyslipidemia, overweight, carbohydrate intolerance, diabetes, hepatic disease, etc.).
- Provide information about the need for long-term multidisciplinary monitoring (endocrinological, cardiovascular, ENT, dermatological, etc.).
- Provide information about the possibility of learning difficulties and probems with social integration (not present in all patients) being related to TS, but point out that intellectual capacity is generally normal, and suggest an assessment, if necessary.
- Cardiac ultrasound and/or cardiac MRI without contrast, to screen for possible malformations, and EKG (measurement of QT). A list of contraindicated drugs should be provided by the cardiologist if a long QT is detected.
- Renal ultrasound to screen for possible malformations.
- ENT consultation with an audiogram assessment of hearing.
- A dental panoramic X ray should be performed in cases of prognathism or temporomandibular joint disorder.
- Bone densitometry (BMD adjusted for patient height) and assessment of calcium and vitamin D intake in food.
- Pelvic ultrasound, with measurement of the size of the uterus (screening for uterine hypoplasia), endometrial thickness, size of the ovaries if visible and antral follicle count. Screening for gonadoblastoma if Y material is detected.
- Blood samples for the following determinations:
  - FSH, LH, estradiol, AMH;
  - Antithyroid Ab (anti-TPO), TSH, ± FT4;
  - HbA1c ± blood glucose and insulin in the fasting state;
  - Cholesterol (total, HDL, LDL), triglycerides in the fasting state;
  - AST, ALT, gamma GT, ALP;
  - Antitransglutaminase (IgA) Ab;
  - Blood creatinine and screening for microalbumin in the urine in cases of renal abnormalities or hypertension;
  - 25(OH)D [25-hydroxy vitamin D].
- Karyotyping or FISH analysis on sex chromosomes if the first karyotyping analysis was performed more than 20 years ago. Screening for Y chromosome material on blood lymphocytes, and/or buccal cells or urine. Molecular analysis is indicated to detect Y-chromosome sequences in TS patients, regardless of their karyotype, but there is no need to check for Y chromosome material in patients with structural abnormalities, such as isoXq or Xp, in the absence of mosaicism.
- Psychological therapy should be suggested.
- Personalized assistance should be suggested in the event of socioprofessional difficulties.
- A visit to the social worker, and if necessary, the MDPH should be suggested.
- The patient should be advised to contact patient organizations.

The diagnostic examinations are described in “Appendix 2”.

## Therapeutic care

### Objectives

- Therapeutic education for patients and/or parents.
- Screening and treatment of associated malformations and comorbid conditions.
- Optimization of growth, induction of puberty, HRT.
- Multidisciplinary management.
- Evaluation of the psychological impact of TS and its consequences for learning and socioprofessional integration.
- Improving the quality of life for both pediatric and adult patients.

### Professionals involved

Multidisciplinary management of the patient can be coordinated by the endocrinology specialist, in a context of short-term hospitalization, day hospitalization, or during consultations at a reference or expert center or locally but in collaboration with an expert center.

- Referral specialist: pediatric endocrinologist, hospital pediatrician experienced in TS, adult endocrinologist, gynecologist.
- Other physicians: cardiologist, ENT specialist, clinical geneticist, general practitioner, cytogeneticist, nephrologist, orthopedist, rheumatologist, gastroenterologist, hepatologist, stomatologist/oral medicine specialist, ophthalmologist, dermatologist, psychiatrist or child psychiatrist, reproductive medicine doctor, plastic surgeon.
- Other health professionals: psychologist, educational therapy nurse, speech therapist, social worker and dietetician (if necessary),
- The modes and frequency of follow-up by these professionals depend on disease severity.

### Therapeutic education

Therapeutic education should include assessments and the dissemination of knowledge about Turner syndrome and its treatments, to ensure that patients and their relatives fully understand and adhere to regular and specially adapted management [3].

It requires coordination between various health professionals working in the hospital environment or locally: doctor, nurse, dietitian, psychologist, speech therapist, psychomotor therapist, physiotherapist, occupational therapist, etc.

This educational activity may take the form of individual consultations or group education organized according to age (prevention of excessive weight gain, psychological aspects, professional integration, etc.), during a consultation or private clinic visit, or within the framework of a day hospital.

Therapeutic education should focus, in particular, on the following points:

- The experience of pediatric or adult patients and their families as concerns TS, its treatments, and its complications, the difficulties encountered, and the impact on the life of the patient and her family.
- Knowledge about TS and its various clinical forms, ensuring that clear and accurate information is imparted, to promote the development of self-care skills and improvements in adherence to treatments and long-term multidisciplinary follow-up.
- Prioritizing the skills to be acquired.
- Pharmacological treatments:
  - Education about growth hormone treatment during childhood, and hormone replacement therapy of the estrogen-progestogen type during adolescence and adulthood;
  - Information about the various treatments/drugs, the reasons for taking them, and their potential adverse effects at any age;
  - Information about the importance of good treatment adherence and screening for potential complications;
  - Information about the possibility of pregnancy with or without MAP.
- Lifestyle:
  - Maintaining a healthy weight (dietetic education, regular physical activity);
  - Implementation of a suitable diet in cases of carbohydrate intolerance or diabetes, celiac disease, dyslipidemia, overweight; appropriate calcium and vitamin D intake;
  - Promotion of good integration into school and the workplace;
  - Referral for psychological, psychomotor, or speech therapy, as appropriate.

### Patient associations

- Health professionals and patients must be informed of the existence of patient associations, reference centers or centers of excellence specializing in rare endocrine diseases, institutional websites, and Orphanet.

Patient associations help to improve the overall management of Turner syndrome by working with patients, their families and caregivers. These groups can help improve the healthcare pathway, building on recognized care networks, and can make families aware of the importance of continuous follow-up at an expert center. They promote the creation of links between patients and families, easing isolation, and ensuring a day-to-day presence and continuous family monitoring. They implement local and national actions in conjunction with health professionals (thematic workshops, national and regional days, support for patient therapeutic education programs, etc.). The institutional websites (www.orpha.net; crmerc.aphp.fr; firendo.fr in France) also provide useful information.

### Therapeutic care

#### Growth hormone (rhGH) treatment

**Short stature** affects about 95% of patients, with adult heights spontaneously reaching values about 20 cm lower than the average for other women of the same ethnic origin and target parental height [4]. The decrease in height velocity is progressive, generally beginning at the age of 18 months. In some cases, an absence of puberty, and therefore of a pubertal growth peak, linked to ovarian insufficiency may be the only warning sign, although this sign is associated with short stature. The efficacy and safety of growth hormone treatment has been demonstrated in clinical trials performed since the 1990s [5–10].

**Treatment with rhGH**

- Is recommended, to improve spontaneous height prognosis and, therefore, the adult height of patients.
- Should be prescribed by a hospital doctor with the necessary authorization, when the patient’s height is ≤ -2 SD or in the event of a significant decrease in height velocity regardless of age, once the diagnosis has been made. The youngest age at which treatment can be initiated remains a matter of debate. In cases of early diagnosis, at a young age, treatment can be started as soon as the growth retardation becomes apparent. However, published studies have yielded conflicting results concerning the effect on adult height of starting treatment before and after the age of four to six years [11].
- The recommended dosage scheme for rhGH is 0.045–0.050 mg/kg/day, as a daily subcutaneous injection administered in the evening.
- Information on the adverse effects of treatment with rhGH should be provided: pain at the injection sites, headache with benign transient intracranial hypertension, peripheral edema, joint pain, carbohydrate intolerance or even diabetes, orthopedic manifestations (epiphysiolysis of the hip, worsening of scoliosis) [12–15]. There is no increase in the risk of developing a brain tumor on growth hormone treatment [16, 17].
- Assessments should be performed, before treatment, for HbA1c ± blood fasting glucose (± OGTT if risk factors: overweight, family history), serum IGF-I concentration, bone age.
- During rhGH treatment, the following should be monitored: IGF-1 levels every 12 months, HbA1c ± blood glucose per year; bone age every two to three years.
- Adaptation of treatment with a scaling back of rhGH dose if IGF-1 level >  + 2.5 SD for at least six months.
- If growth rate is insufficient on rhGH (HV < 3 cm/year), then possible injection technique errors, poor compliance, hypothyroidism, celiac disease, inflammatory bowel disease, or pernicious anemia should be investigated.
- Treatment with rhGH should be stopped once puberty is complete and growth rate is below 2 cm/year.

#### Ovarian insufficiency treatment

**Premature ovarian insufficiency** is defined as menstrual cycle disorders (amenorrhea or abnormally short cycles) persisting for over four months together with FSH levels exceeding 25 mIU/ml in two determinations, before the age of 40 years. It affects 95% of patients with TS. A spontaneous onset of puberty is observed in up to 30% of patients (particularly in forms with 45,X/46,XX mosaicism), with spontaneous menstruation in 10–20% of cases. In most cases, this ovarian activity ceases within a few years: puberty stops progressing, and primary or secondary amenorrhea occurs. Spontaneous pregnancies have been reported in 7% of cases, especially in cases of 45,X/46,XX mosaicism [18].

Patients with spontaneous ovarian activity should be monitored, to determine whether or not secondary hormone replacement therapy is useful [19]. In all other cases, puberty should be induced by hormone replacement therapy.

Various protocols involving different drugs and galenic formulas, and various doses are used, depending on the prescribing practices of each center, and efficacy is often similar for these different protocols, regardless of the galenic formula used. The percutaneous route is often used for pubertal induction. This route has the advantage of reducing first-pass hepatic metabolism and causing less disruption of coagulation than oral administration [20–22]. This route is therefore preferred for adult patients with a history of venous thromboembolic disease, migraine with aura or high cardiovascular risk [23]. The aim is to keep delivering low doses of estrogen until growth drops off, the doses used being sufficient to induce puberty in a satisfactory manner, without inducing a rapid progression of bone maturation.

In adults, in the absence of very rare contraindications (hormone-dependent cancers), hormone replacement therapy should be maintained at least until the physiological age of menopause (around 50 years), with annual reassessment by the doctor for each individual. The main goals of HRT are the prevention of cardiovascular and bone risks and the improvement of sexual function.

- Pediatric treatment of ovarian insufficiency. The need for puberty induction and the possibility of infertility should be discussed.
- Treatment with estrogen to induce puberty is generally initiated at the age of about 11 to 12 years and/or at a bone age of about 11 years, when FSH levels are high (> 10 IU/L). In the rare cases in which puberty begins spontaneously, it is important to monitor its pace and ovarian function with clinical and laboratory examinations, including determinations of serum estradiol, FSH and LH and annual determinations of AMH, to identify patients in whom puberty is failing to progress, who may require hormone replacement therapy (“Appendix 5”).
- Pre-treatment assessment for estrogen:
  - During the medical interview, the doctor should check for risk factors for thromboembolic disease (history of thromboembolic disease in a first-degree relative), history of liver disease or dyslipidemia, BP should be checked after 10 min of rest, preferably in both arms and at least in the right arm (especially if there is a history of aortic coarctation), to assess the vascular risk;
  - Thrombophilia should be assessed in patients with a family history of thromboembolism in a first-degree relative (with the relative concerned tested first) and/or a personal history of venous thromboembolism;
  - Assays for estradiol, FSH, LH, AMH (anti-Müllerian hormone, residual ovarian activity) should be performed;
  - Triglycerides, total cholesterol, and fasting HDL and LDL determinations in the fasting state, should be performed at the end of puberty, and then between the ages of 17 and 21 years, or earlier, if the patient has risk factors (overweight, family history of dyslipidemia);
  - Liver function tests (AST, ALT, GGT, ALP).
- The optimal age, dose, type, and sequence have yet to be clearly defined for estrogen treatment. The starting dose is usually about one tenth the adult estrogen replacement dose (i.e. 0.2 mg/day 17-beta-estradiol), preferably administered percutaneously, and as 17-beta-estradiol, not ethinylestradiol [24].
- In late-diagnosed forms, it may be preferable to wait for one year of treatment with GH to have elapsed before starting estrogen therapy, to optimize prepubertal growth on GH.
- Treatment with low-dose estrogen should be continued for at least two years, to allow optimal breast and uterine development and to prevent an excessive progression of bone maturation. At the end of the growth period (growth rate < 2 cm/year, and/or cartilage fusion) estrogen dose is scaled up to the adult replacement dose and progestogens are introduced.
- Recent clinical guidelines recommend monitoring hormonal substitution through determinations of serum estradiol, FSH and LH. Estradiol levels are positively associated with bone mineral density. However, the target estradiol levels required for optimal substitution have yet to be determined [25].
- A progestogen can be added, once estrogen dose has reached 1 mg 17-beta-estradiol/day, or in the event of bleeding onset on low-dose estrogen alone. Discontinuous estrogen-progestogen treatment allows menstruation to begin, generally at the end of the 1st cycle on estrogen-progestogen replacement therapy [26]. The use of 200 mg natural progesterone or 10 mg dydrogesterone is preferable to the use of synthetic progestins for hormone replacement therapy, because of the greater risk associated with the long-term use of synthetic progestogens recently reported for the general population [23, 27, 28].
- Estrogen-only treatment should be maintained for no more than two to three years before the introduction of progestogens. In some cases, sequential treatment with progesterone/progestogens only (10 days/month) may be introduced after menarche, if puberty begins spontaneously, but with an abnormal duration of menstrual cycles. Natural progesterones should be preferred, again to reduce the risk of meningioma associated with the intake, duration and dose of certain progestogen treatments (Cf. ANSM 2021, https://ansm.sante.fr/actualites/lutenyl-luteran-des-documents-pour-garantir-linformation-des-femmes-sur-laugmentation-du-risque-de-meningiome).
- If hypogonadism is identified after puberty, the initial phase of estrogen treatment may be shortened to six months, but the increase in dose must remain very gradual to ensure correct breast development.
- Girls with TS should be offered a consultation with a gynecologist in addition to the vaccination against papillomavirus and hepatitis B offered to all girls. Adjustments of estrogen treatment may be monitored in collaboration with the gynecologist.
- The presence of Y chromosome material in the cytogenetic study, and/or FISH analysis should lead to further investigations as to whether the region of susceptibility to GBY gonadoblastoma (the gonadoblastoma locus on the Y chromosome) is present (on a partial or entire Y chromosome). Gonadectomy, and fertility preservation by freezing ovarian tissue, depending on residual ovary function, should be discussed on a case-by-case basis, at a multidisciplinary coordination meeting (MCM).
- Fertility preservation should be discussed as a function of age (over 10 years) and residual ovary function.
- Contraception should be discussed according to residual ovary function.

**Treatment of ovarian insufficiency in adulthood**

- Continuation (or initiation in cases of late diagnosis) of estrogen-progestogen therapy (“Appendix 5”). Treatment should be initiated only after evaluation of the various risk factors, to ensure the choice of the most suitable route of administration.
- Pre-treatment assessment for hormone replacement therapy:
  - During the medical interview, the doctor should check for risk factors for thromboembolic disease (family history of thromboembolic disease in a first-degree relative), history of liver disease, cardiovascular risk factors, such as dyslipidemia, diabetes, and high BP;
  - Thrombophilia should be assessed in patients with a family history of thromboembolism in a first-degree relative (with prior evaluation of the relative concerned) and/or a personal history of venous thromboembolism;
  - If necessary, assays for estradiol, FSH, LH, and AMH (residual ovarian activity) should be performed in patients with no HRT for at least two months;
  - Lipid panel, cholesterol (total, HDL, LDL) and triglycerides should be determined (especially if treatment with estrogen-progestogen contraception is planned);
  - Liver function tests (AST, ALT, GGT, ALP) should be performed.
- In most cases, the treatment administered is 17-beta-estradiol at a dose of 2 mg/day, combined with progesterone or, more rarely, a progestogen for at least 12 days per month in the case of discontinuous estrogen therapy and 14 days per month in the case of continuous estrogen therapy, to prevent excessive endometrial proliferation. Different dosage regimes are possible, depending on whether or not the patient prefers to have periods during the withdrawal period, and on the patient's estrogen deficiency symptoms during the non-treatment phases (“Appendix 5”).
- An estrogen-progestogen treatment containing ethinylestradiol can be prescribed in some cases, provided that the contraindications for the general population are respected (HAS 2019: contraception in women at cardiovascular risk, and contraindication in the event of a personal history of hormone-dependent cancer). However, despite the better compliance sometimes achieved with this treatment, it may be less favorable than treatment with 17-beta-estradiol in terms of its effect on bone mineralization and carbohydrate-lipid metabolism, and it also increases vascular risks.
- From the age of 35–40 years, estrogen-progestogen therapy including ethinylestradiol, if prescribed, should be replaced by hormone replacement therapy containing 17-beta-estradiol, preferably delivered percutaneously and combined with progesterone or a progestogen, to minimize cardiovascular and venous thromboembolism risks.
- If there is a risk of venous thromboembolism or cardiovascular risk (hypertension, overweight, diabetes, migraines with aura, smoking and a prior history venous thromboembolic disease), transdermal 17-beta-estradiol treatment is preferable to the oral administration of this molecule and to the use of ethinylestradiol.
- HRT has beneficial effects on somatic and psychological wellbeing, by ensuring an appropriate adult phenotype with respect to breast, vagina-uterine and bone development, body composition, metabolic health, the cardiovascular system, cognitive functions, and the patient’s sex life, all of which should be explained to the patient, to promote treatment compliance [29–31].
- Information on the importance of continuing estrogen-progestogen therapy at least until the physiological age of menopause (around 50 years) should be provided.
- In the event of liver disease, neither route of administration (oral or transdermal) is contraindicated [32]. In the event of uncontrolled celiac disease, the transdermal route should be preferred (malabsorption).
- Patients with spontaneous cycles should be informed of the need for contraception. It is important to discuss family planning issues, because of the risk of premature ovarian insufficiency. If there are no routine contraindications for estrogen-progestogen contraception, then such contraception can be prescribed.
- Gynecological follow-up in adulthood is identical to that for the general female population (cervical smears, mammograms, etc.). The risk of breast cancer is not increased by HRT.
- Information should be provided about the possibility of pregnancy, either spontaneous (7%) or, much more frequently, through MAP (egg donation), in the absence of cardiovascular contraindications.
- In patients with spontaneous cycles, fertility preservation should be considered, depending on the results of analyses of the ovarian reserve, including determinations of serum estradiol, FSH, LH and AMH. Ovary function should be monitored annually.
- All pregnancies in TS patients should be considered at high risk of complications, and patients should be referred to a level 2 or 3 maternity hospital.
- Information should be provided about the need for pre-conception analyses and regular cardiovascular monitoring throughout the pregnancy (BP after 10 min of rest, preferably in both arms, and at least in the right arm (especially if there is a history of aortic coarctation), aortic diameter by imaging). The most recent cardiovascular imaging data available should be less than two years old at the time of conception.

#### Other treatments

**Dietary and lifestyle measures, medicinal and non-surgical treatments, if required**

- Dietary and lifestyle recommendations (balanced diet, with low levels of simple sugars if the patient has carbohydrate intolerance or diabetes).
- Treatment of diabetes (oral antidiabetic drugs and/or insulin, depending on whether the underlying mechanism is autoimmune).
- Treatment of dysthyroidism (levothyroxine, anti-thyroid drugs if required).
- Antihypertensive treatment, with beta-blockers if the patient presents aortic dilation.
- A gluten-free diet for celiac disease.
- A low-fat diet in cases of dyslipidemia, with drug treatment.
- Appropriate treatment in the event of liver disease.
- Physiotherapy, psychomotor skills training, speech therapy.
- Orthodontic treatment.
- Hearing aids and glasses.
- Lymphatic drainage, with the wearing of compression stockings, nightly compression wraps.
- Vitamin D and calcium supplementation.
- Dietary and lifestyle counseling (balanced diet, regular physical exercise).
- Appropriate treatment in the event of skin problems.

### Surgical treatments (if required)

- Cardiac/vascular surgery (coarctation, high-risk aortic dilation, etc.).
- ENT surgery (fitting of transtympanic ventilation tubes, cholesteatoma, etc.), stomatology.
- Ophthalmological surgery (in cases of strabismus, for example).
- Urological surgery (in cases of vesicoureteral reflux, or if there are complications resulting from a urological malformation).
- Plastic surgery (hypoplasia or abnormal breast development, pterygium colli/web neck, controversial due to the risk of keloids).
- Orthopedic surgery or use of an orthopedic device in the event of severe scoliosis.
- Appropriate surgery in cases of cancer (mostly of the skin).
- In patients with Y chromosome material, the indication and appropriate age for prophylatic gonadectomy should be discussed at an MCM, taking into account the theoretical risk of malignant transformation (gonadoblastoma with risk of dysgermioma) in the longer term.

## Follow-up

### Objectives

- To screen and manage possible associated diseases/conditions.
- To monitor treatment efficacy, tolerance and compliance.
- To assess regularly the knowledge of the patients and their families concerning TS.
- To continue the therapeutic education of patients and their families.
- To assess the social impact and provide support if necessary: academic/learning, socioprofessional support; identification of any possible disability, application for formal ALD status.
- To ensure that the general practitioner and other specialists are kept informed (multidisciplinary follow-up care).

### Professionals involved

The various professionals involved in follow-up care and monitoring have already been listed in the sections concerning the initial assessment and therapeutic management.

### Frequency of consultations

- In all cases, at a reference or expert center for rare endocrine diseases, or locally but in collaboration with such a center:
- In children: in the absence of treatment with rhGH or other associated diseases, patients should be assessed annually. Assessments should take place every six months for patients on rhGH treatment.
- In adults: patients should be seen every six to 12 months, depending on the possible associated diseases and how frequently follow-up is considered to be needed.
- If adult patients are followed locally (by an endocrinologist, medical gynecologist), a complete assessment should be performed at a reference or expert center every one to five years (depending on associated conditions).
- Methods:
  - Regular follow-up should take place at clinics, day hospitals, or, more rarely, during a period of short-term hospitalization;
  - Certain supplementary examinations may occasionally be performed locally;
  - Between specialist visits, the general practitioner will treat any other conditions that arise, if necessary, in partnership with a doctor from the reference or expert center.

### Content of consultations

A summary table of the follow-up to be performed, as a function of patient age, for TS management, is available in “Appendix 3”.

#### At each visit

- A clinical examination should be performed, assessing weight, height, puberty stage, BMI, BP after 10 min of rest, preferably in both arms, or at least in the right arm (especially if there is a history of aortic coarctation), and the patients should be checked for heart murmur.
- Growth should be monitored during the pediatric period (standard and Turner curves).
- During the medical interview, the doctor should check for functional signs of dysthyroidism, heart issues, digestive issues, bleeding on HRT, menstrual cycle disorders, estrogen deficiency, hearing difficulties, etc.
- Compliance with treatments should be checked, together with potential adverse effects.
- Academic learning should be evaluated.
- Lifestyle, including physical activity, professional activity, and social relations should be evaluated.
- The patient’s psychological condition should be assessed.
- The patient’s knowledge of the disease and of the treatments received, and of the importance of screening for complications, depending on the patient's age should be evaluated.

#### If necessary

- A consultation may be organized with a nurse educator specializing in endocrinology.
- A consultation with a dietitian (overweight, carbohydrate intolerance, diabetes, dyslipidemia, celiac disease) may also be organized.
- A consultation should be organized with a psychologist, with assessment of TS experience and self-esteem, and neurocognitive assessment, if necessary.
- The patient may be assessed by a psychologist, to obtain assistance with psychomotor skill development, and an assessment of the possible need for learning support.
- A consultation with a social worker may be organized.
- Specialist clinics (gynecology, genetics, ENT, cardiology, hepatology, ophthalmology, stomatology/oral medicine, orthopedics, etc.) may be attended by the patient.

#### Regular therapeutic education

- There should be regular assessments and updating of knowledge about TS, appropriate for the patient's age (answering the patient’s questions, refining the diagnosis).
- During pediatric care, it is important to ensure that patients are progressively given information appropriate for their age, particularly during pre-adolescence and adolescence, and to assess their knowledge and perception of TS. The knowledge of the patient’s parents should also be checked.
- Short stature, premature ovarian insufficiency, and screening for potential associated diseases should be progressively explained to the patient in an age-appropriate manner.

#### Transition from pediatric to adult care

- There should be a gradual preparation towards the end of adolescence, for the transition from pediatric services to an adult endocrinology structure providing appropriate multidisciplinary care. The optimum age for this transfer has yet to be determined, but it generally occurs at about 16 to 18 years of age. Pediatricians are responsible for ensuring the continuation of their patient’s care in adult services.
- Doctors should identify adult endocrinology facilities offering multidisciplinary care and capable of ensuring a continuity of follow-up for any diseases diagnosed during childhood and detecting any potential associated disorders during adulthood.
- The pediatric medical file should be sent to the adult service: a summary sheet (“Appendix 4”).

#### First consultation in adult services

TS is mostly diagnosed during childhood. The first consultation with adult healthcare services provides an opportunity:

- To perform a clinical examination, determining weight, height, BMI, BP after 10 min of rest, preferably on both arms, and at least on the right arm (especially if there is a history of aortic coarctation), and to check for heart murmur, secondary sex characteristics, and morphological particularities. The medical interview provides an opportunity to check for functional signs of possible associated diseases (cardiac, digestive, menstrual cycle disorders or estrogen deficiency, dysthyroidism, hearing difficulties, etc.).
- To evaluate quality of life, lifestyle, including physical activity and socioprofessional integration,
- To evaluate the patient’s knowledge and experience of TS.
- To stress the importance of long-term multidisciplinary follow-up.
- To stress the importance of long-term hormone replacement therapy, in cases of premature ovarian insufficiency, at least until the physiological age of menopause (50 years), and of other treatments in general, explaining the consequences of not taking treatments correctly.
- To discuss the possibility of fertility preservation in the event of sustained ovarian function (egg freezing) and the possibility of MAP for planned parenthood (in vitro fertilization with egg donation).
- To collect all the clinical and paraclinical data from pediatric follow-up:
  - Circumstances and age at diagnosis, karyotyping results, ages at the start and end of GH treatment, induced or spontaneous onset of puberty, associated diseases and known complications;
  - List of treatments (estrogen and progestogens, thyroid hormones, antihypertensive drugs, anti-diabetic agents, etc.), checks for potential side effects and possible reasons for stopping treatment;
  - Dates and results of the most recent laboratory tests and morphological examinations.
- To write a summary sheet for adult follow-up.
- To offer the possibility of a consultation in the presence of the patient’s spouse/partner, to allow the couple to discuss the repercussions of TS.

### Pregnancy management

Spontaneous pregnancies are rare [33]. However, in the event of pregnancy, whether spontaneous or after egg donation:

- Preconception analyses can be performed, if desired. In all cases, if the patient becomes pregnant, the following analyses should be performed:
  - Cardiovascular: BP after 10 min of rest, preferably in both arms, and at least the right arm (especially if there is a history of aortic coarctation), EKG, precise evaluation of aortic diameter indexed to body surface area (BSA) (ultrasound scan performed by an expert practitioner, or even MRI);
  - Anti-TPO Ab, TSH, FT4, fasting blood glucose, HbA1c, and creatinine determinations;
  - Pelvic ultrasound scan, to be performed before any pregnancy is planned.
- The pregnancy should be considered at high risk of complications and the patient should be referred to a level 2 or 3 maternity unit with the possibility of transfer to a nearby cardiothoracic surgery unit [34, 35].
- Fasting blood glucose levels should be assessed in the first trimester of pregnancy, and carbohydrate tolerance (OGTT) should be assessed in the second trimester of pregnancy (if fasting blood glucose levels in the first trimester are normal).
- The patient should be referred to a specialist cardiologist for cardiovascular monitoring throughout the pregnancy: BP measurement at each visit, measurement of aortic diameter by ultrasound at the end of the 1st and 2nd trimesters, and monthly during the last trimester, with a check-up 8 to 15 days postpartum (possibly with aortic MRI, if the specialist cardiologist believes it to be warranted) [36].
- Information should be provided about the higher than average risk of miscarriage, and of chromosomal abnormalities (gonosomes) that may occur in spontaneous pregnancies, with the possibility of antenatal diagnosis.
- The doctor should ensure that the patient is aware that there is a higher risk of cardiovascular complications, prematurity and IUGR, and the possibility of a cesarean section.

## Screening and management of associated disorders

### Endocrine conditions

Some autoimmune diseases are more frequent in patients with Turner syndrome than in the general population: autoimmune thyroiditis, celiac disease, type 1 diabetes, and, more rarely, Graves' disease, and pernicious anemia [37–41]. Both types of diabetes (1 and 2) can be observed in TS patients. Studies on carbohydrate tolerance have shown that the risk of glucose intolerance and diabetes is moderately increased by TS, regardless of GH or estrogen treatment [42]. GH treatment can induce insulin resistance, which is generally reversed when treatment is stopped.

#### Thyroid conditions

- Anti-TPO Ab, TSH ± FT4, assessment at diagnosis in patients aged at least four years, and annually thereafter.
- Thyroid ultrasound in cases of dysthyroidism, to check for thyroid nodules and/or goiters.
- L-thyroxine replacement therapy in cases of hypothyroidism, with consultation and monitoring of serum TSH and FT4 every six months in pediatric care and then every six to 12 months during adulthood.
- Treatment with synthetic antithyroid drugs in cases of Graves' disease, with laboratory tests performed every three to four months.

#### Carbohydrate intolerance and diabetes

- Information should be provided about the high risk of carbohydrate intolerance or even diabetes.
- During the medical interview, patients should be asked whether they have a family history of diabetes.
- HbA1c ± blood glucose should be monitored annually from the age of 10 years (diabetes if fasting blood glucose concentration ≥ 7 mmol/L in two consecutive determinations and 11 mmol/L at any time of day; moderate fasting hyperglycemia if blood sugar concentration is between 5.5 mmol/L and 6.9 mmol/L).
- An OGTT should be performed in cases of moderate increase in HbA1c (between 5.8 and 6.4%) and/or fasting blood glucose concentrations (between 5.5 and 6.9 mmol/L) during childhood, before treatment with GH in patients with risk factors (overweight, family history) and systematically in pregnant patients.
- Dietary and lifestyle measures should be implemented in the event of carbohydrate intolerance (blood glucose concentration at 120 min of between 7.7 and 11 mmol/L in an OGTT) or diabetes (a balanced diet low in sugar, moderate-to-heavy physical exercise for at least 30 min per day).
- Systematic screening for autoantibodies (anti-beta cells, anti-islets of Langerhans, anti-GAD65, anti-IA2, anti-ZnT8) should be performed in the event of impaired carbohydrate tolerance/diabetes.
- Appropriate drug treatment should be initiated in cases of diabetes (oral antidiabetic agent and/or insulin treatment depending on whether the condition is autoimmune).
- HbA1c should be monitored every three to four months in cases of diabetes, at any age.

### Celiac disease

- Signs suggestive of celiac disease (weight loss, abdominal pain, diarrhea, insufficient growth on rhGH treatment, iron deficiency anemia, other signs of malabsorption) should be sought.
- Screening should be performed by means of antitransglutaminase IgA assays at diagnosis (with total IgA determination to eliminate IgA deficiency) in patients aged at least two to three years, then every two years thereafter during pediatric care, and every three years during adulthood, or at a shorter interval in the event of symptoms.
- In cases of IgA deficiency, an antitransglutaminase IgG assay should be used to test for celiac disease, not an antitransglutaminase IgA assay.
- In accordance with the recommendations of the ESPGHAN (European Society for Pediatric Gastroenterology, Hepatology and Nutrition), the diagnosis of celiac disease can be based on clinical and biological criteria only if all of the following criteria are met: suggestive symptoms, antitransglutaminase IgA levels more than 10 times higher than normal and positive for anti-endomysium IgA. In all other cases, an upper GI endoscopy with duodenal biopsy is required to confirm the diagnosis.
- HLA class II genotyping (for an HLA-DQ2 or -DQ8 screen) may be considered with a pediatrician/gastroenterologist, in cases of doubt concerning the diagnosis, or to determine the benefits of repeat screening (in the absence of HLA-DQ2 or -DQ8, the patient is very unlikely to have developed celiac disease and repeat screening is not, therefore, required).
- Specialist care should be provided for cases of confirmed celiac disease.

### Cardiovascular management

Congenital heart defects are found in about 50% of patients, the two most frequent defects being bicuspid aortic valve (BAV) (about 30%) and aortic coarctation (about 15%) [43–45]. Venous malformations are rare [46].

The following acquired and potentially severe cardiovascular complications may be observed: hypertension, aortic dilation (aneurysm) with a potential risk of aortic dissection, more rarely, atherosclerosis and cerebrovascular accidents [47–56]. There is also a high risk of hypertension (HT) from childhood onwards, and HT is reported in about half of all adult patients [57–61]. The main objective of management is to limit aortic dilation and the risk of dissection. EKG abnormalities (conduction or repolarization disturbances: right-axis deviation, T-wave abnormalities, accelerated atrioventricular conduction, QT interval prolongation) have been reported [62]. Being on growth hormone treatment does not seem to increase the risk of these complications.

#### Screening for congenital heart defects and analysis of the aortic arch

##### Cardiological screening

**Clinical evidence:** The purpose of screening is to make it possible to manage cardiac and vascular complications, such as BAV, dilation of the thoracic aorta, aortic coarctation and HT. The cardiovascular clinical examination should focus on cardiothoracic auscultation to check for aortic valve murmur and bruits (subclavian thrill, posterior murmur), palpation of the peripheral pulses, and blood pressure assessment.

**Paraclinical evidence**

- **Two-dimensional transthoracic doppler echocardiography** (TTE): this is a widely used first-line technique. For more information on this technique, refer to the dedicated NDCP (https://www.has-sante.fr/upload/docs/application/pdf/201211/rapport_eval_ett_octobre_2012_vd.pdf). TTE can be used to check for aortic dilation by measuring diameters at reference levels: aortic ring, aortic sinus, sinotubular junction, tubular ascending aorta, aortic arch, descending thoracic aorta *Children*: before the age of 16 years, the published Z-scores available are those of Quezada et al. [63]. *Adults*: after the age of 16 years, the published standards are those of Roman et al*.* [64], which are dependent on body surface area (BSA) and age. The normal progression of adult aortic diameter with age in women is + 0.7 mm every 10 years [61].
- **MR angiogram slices (or angio-CT scan):** *Children:* This type of imaging is recommended if there is any doubt or incomplete visualization of the entire aorta on ultrasound scans performed to check for BAV, aortic coarctation or aortic dilation. *Adults:* Imaging is recommended at baseline, to provide visualization of the entire aortic arch, ascending and descending aorta, and for screening for other vascular abnormalities. It can also be used to check the dimensions measured on ultrasound scans, and before surgery or conception. Aortic dilatation is diagnosed if the diameter of the ascending tubular aorta indexed to BSA (body surface area) (aortic index) exceeds 20 mm/m^2^ in patients over the age of 16 years.

##### Subsequent cardiological follow-up

**Clinical evidence**

Monitoring should be tailored to the patient, especially in the presence of risk factors for congenital or acquired aortic dilation (BAV, coarctation or HT) and depends on aortic diameter, or changes in this diameter. It should be performed in collaboration with cardiologists trained in congenital aortopathies. After HT diagnosis with a 24-h BP Holter, blood pressure should be effectively controlled, with a BP treatment goal of 130/80 mmHg. Any thoracic symptoms in patients known to have advanced aortic dilatation should prompt rapid contact with the patient’s cardiologist.

**Paraclinical evidence**

*Children:* Indexing aortic diameter to body surface area gives an overly high aortic index in children. Use of the dedicated Z-score for the Turner syndrome population is therefore recommended. Aortic dilation is rare during childhood and is defined as a Z-score of ≥ 3 SDS. However, measurements should be adapted by a cardiac pediatrician trained in the management of Turner syndrome.

*Adult:* A TTE should be carried out in case of HT diagnosis. In the absence of a specific study comparing TS patients with adult women of the same height without TS, it is difficult to define an at-risk aortic diameter threshold. According to published findings, aortic diameter values exceeding 33 mm for the aortic sinus and 31 mm for the ascending aorta are considered, by some, to be pathological [65]. For a mean body surface area of 1.6 m^2^, an indexed ascending aortic diameter > 25 mm/m^2^ corresponds to a diameter of 40 mm, i.e. a Z-score specific for Turner syndrome > 4 SDS. In overweight patients, the absolute value for aortic diameter can be used.

**Monitoring rate**

*Childhood (after diagnostic investigations):*

- LOW RISK—in the absence of risk factors (defined as a BAV, aortic coarctation or its equivalent, an elongated transverse aortic arch, HT) and with an aortic Z score ≤  + 3 SDS: ultrasound monitoring every five years, and then systematically at the end of adolescence, before the transition to adult care.
- MODERATE RISK—in the absence of risk factors and with an aortic Z score ≥  + 3 SDS: annual ultrasound monitoring by the pediatric cardiologist, and then systematically at the end of adolescence, before the transition to adult care.
- MODERATE RISK—with risk factors but with an aortic Z score ≤  + 3 SDS: ultrasound monitoring every one to two years by the pediatric cardiologist, then systematically at the end of adolescence, before the transition to adult care.
- HIGH RISK—in the presence of risk factors and with an aortic Z score ≥  + 3 SDS: ultrasound monitoring every six months to one year by the pediatric cardiologist, then at the end of adolescence, systematically, before the transition to adult services.

*Adults (after assessment at diagnosis):*

- LOW RISK—without risk factors (no BAV, no dilation, no HT) and with an aortic index ≤ 20 mm/m^2^: transthoracic ultrasound monitoring or cardiac and aortic MRI every 5 to 10 years.
- MODERATE RISK—in the absence of risk factors and with an aortic index of 20 to 23 mm/m^2^: ultrasound/MRI monitoring every 3 to 5 years.
- MODERATE RISK—with risk factors (BAV, HT in particular) but with an aortic index ≤ 23 mm/m^2^: ultrasound/MRI monitoring every 2 to 3 years.
- MODERATE RISK—with risk factors and an aortic index ≥ 23 mm/m^2^: annual ultrasound/MRI monitoring.
- HIGH RISK—with risk factors and with an aortic index > 23 mm/m^2^: MRI monitoring every six months to one year. Follow-up at closer intervals is possible when aortic surgery is considered.

In the event of aortic dilation or BAV, regardless of BP (even if normal), treatment with beta-blockers should be considered and prescribed, if necessary, by the cardiologist.

Given the reported cases of aortic dissection, and by analogy with other aortic conditions, specialist medical and surgical advice should be obtained if the following are present (with or without risk factors):

- Aortic diameter > 40 mm.
- Aortic index > 25 mm/m^2^ (Z score >  + 4 SDS).
- Aortic progression >  + 5 mm/year (Z score >  + 1 SDS/year).

Physical activity (sports) is recommended for patients, but may require adaption according to the presence or absence of aortic dilation and its severity: in the presence of a dilation > 20 mm/m^2^, closed glottis strain should be avoided (e.g. weightlifting), and competitive sports are contraindicated in the event of a dilation > 23 mm/m^2^. The cardiologist should provide a list of contraindicated and authorized sports.

#### Indication for surgical prosthetic replacement of the ascending thoracic aorta

- Prophylactic surgery may be considered in patients under the age of 16 years, if the ascending aorta has a Z score ≥ 4 SDS, with or without associated risk factors for dissection (bicuspid aorta, elongated transverse aortic arch, aortic coarctation and hypertension).
- Prophylactic surgery is indicated in patients aged 16 years and over with an indexed aortic diameter ≥ 25 mm/m^2^ associated with risk factors for dissection.
- Prophylactic surgery may be considered in patients aged 16 years and over with an indexed aortic diameter ≥ 25 mm/m^2^ but no risk factors for dissection.

#### Cardiovascular monitoring

- **At the time of TS diagnosis**, a consultation with a pediatric cardiologist or cardiologist specializing in adult congenital heart diseases is necessary. The frequency of any subsequent cardiological follow-up depends on whether there are any malformations or associated cardiovascular disorders. Echocardiography should be performed to screen for BAV and for aortic arch explorations (+ MRI in adults during diagnostic evaluations). Subsequent monitoring rate: see above.
- **An assessment of QT** by EKG should be performed at baseline, with subsequent check-ups, as recommended. If a prolonged QT interval (> 460 ms; calculated with Hodge's formula, taking the presence of tachycardia into account) is detected, a cardiological examination with 24-h Holter EKG and stress EKG is recommended. The cardiologist should provide a list of contraindicated drugs for patients with a long QT.
- **BP monitoring** should be performed annually: if HT is detected, a secondary cause (ultrasound/Doppler scans of the renal arteries) should be sought. ABPM/Holter measurements should be considered if blood pressure values are repeatedly borderline. Antihypertensive treatment should be prescribed, with a therapeutic objective of 130/80 mmHg. By analogy with Marfan syndrome, beta blockers and angiotensin 2 receptor antagonists (ARB 2) can be used. Calcium channel blockers should be avoided because of their vasodilator activity and the risk of lymphedema.
- **Screening for sleep apnea** should be performed in the event of a loss of the nocturnal dip in blood pressure, obesity, and nighttime snoring.
- **Other cardiovascular risk factors** (overweight, smoking, diabetes, dyslipidemia) should be checked, with the a calcium score test performed, if necessary. Regular physical activity should be encouraged.
- **Dental hygiene is important** and antibiotic prophylaxis against endocarditis should be prescribed during risk procedures (invasive dental care, surgery, etc.), in accordance with the recommendations of the cardiologist.

#### In case of pregnancy

Pregnancy may be possible (spontaneous in rare cases, but more usually after IVF with egg donation), but must be planned. **A pre-conception evaluation** is essential, with a clinical examination, auscultation, and strict BP control, with a gestational blood pressure target < 135/85 mmHg, in addition to the systematic testing of thyroid, glucose and liver functions. Cardio-aortic imaging must be performed in the year before a planned pregnancy and/or if a pregnancy is planned. These high-risk pregnancies, which may occur naturally but mostly arise from egg donation, should be discussed on a case-by-case basis at an MCM, to ensure that all the necessary information is available for the authorization of a pregnancy.

**The contraindications** for future pregnancy currently recognized are: a history of aortic valve replacement or aortic dissection, an indexed ascending aortic diameter > 25 mm/m^2^ or > 20 mm/m^2^ in the presence of risk factors for dilation (BAV, coarctation of the aorta (COA) or elongated transverse arch, uncontrolled HT, and/or liver disease with a risk of gravidic cholestasis.

**During pregnancy**, cardiological monitoring at a reference center with access to a thoracic surgery center is recommended, with a clinical examination, BP measurement and aortic imaging recommended once during each of the 1st and 2nd trimesters of pregnancy, and then monthly during the 3rd trimester, and then within eight days and then two months after delivery [66]. Management, from the start of pregnancy, includes the monitoring of TS-related comorbid conditions, with the checking of thyroid function, glucose and liver panels.

**Pregnancy management** in these patients includes several special features [67, 68]. In the case of egg donation, only one embryo should be transferred, to prevent the aortic hemodynamic stress of a multiple pregnancy. The higher risk of pre-eclampsia may justify the prescription of 75 mg aspirin from the 12th week of amenorrhea onwards, and the close monitoring of BP thereafter. In patients with risk factors (dilation, BAV, HT), more frequent monitoring during and after pregnancy may be necessary. Aortic dilation or gestational hypertension can be treated medically with beta blockers, in collaboration with the cardiologist. Pregnancy and childbirth should be supervised by an upstream multidisciplinary approach including input from anesthesiologists, obstetricians, cardiologists, endocrinologists and pediatricians. If aortic dissection occurs and the fetus is viable, maternal aortoplasty remains the most urgent issue [5]. In such cases, the infant must be delivered by cesarean section. Even in uneventful pregnancies, delivery should be scheduled and should take place at a specialist center at which emergency maternal thoracic surgery could be performed if necessary, regardless of the presence or absence of a neonatal unit.

Vaginal delivery is indicated in TS patients with an indexed ascending thoracic aortic diameter < 20 mm/m^2^ and if the width of the patient's pelvis allows. In the event of an aortic index of 20 to 25 mm/m^2^, a vaginal delivery with epidural anesthesia may be considered, but the patient must be informed of the possibility of conversion to cesarean section. Cesarean delivery is indicated in TS patients with an indexed ascending thoracic aortic diameter > 25 mm/m^2^ [5]. In the postpartum period, a follow-up cardiac ultrasound scan should be performed no later than two months after delivery.

The potential risk of the baby inheriting TS (in cases of an X-structure abnormality) or other chromosomal abnormalities should be discussed; this risk is higher than in the general population for spontaneous pregnancies. In such cases, the possibility of antenatal diagnosis should be discussed (not required in cases of egg donation, and this is not an indication for preimplantation diagnosis).

### ENT management

TS favors acute otitis media, which often occurs between the ages of one and six years, with a peak frequency at about three years of age (hypoplasia and dysfunction of the eustachian tubes, velopharyngeal insufficiency (VPI)), and increases the risk of conduction deafness, which is common in patients with TS.

Sensorineural deafness may occur during childhood. It is common in adulthood (more than 50% of patients between the ages of 15 and 35 years suffer hearing loss) and worsens with age [69–74].

**ENT monitoring and management**

- Information about the risk of hearing loss should be provided.
- Follow-up should include an ENT consultation with otoscopy and hearing tests (screening for deafness) at of the diagnosis of TS, and every two to five years thereafter.
- In the event of an ENT disease/condition arising, appropriate care with more frequent ENT follow-up is recommended.
- Information about the child’s risk of recurrent bouts of otitis, their possible complications and the need for appropriate effective treatment (antibiotics) should be provided, with systematic otoscopic follow-up throughout the otitis episode.
- The insertion of trans-tympanic ventilators should be considered.
- Speech therapy should be prescribed in the event of speech/language problems.
- Appropriate hearing aids should be fitted in cases of hearing loss.
- Patients should be screened for possible sleep apnea.
- Appropriate treatment should be given if necessary: spontaneous ventilation with continuous positive airway pressure (CPAP), a mandibular advancement device, or maxillofacial surgery.

### Nephrological management

Renal malformations are present in about one third of cases (horseshoe kidney, malrotation, ectopic kidney, renal agenesis, duplex kidney) [75–78]. These malformations are of no clinical significance in most cases, but some malformations may cause hypertension, a predisposition to urinary tract infections and, in some cases, hydronephrosis [79].

**Nephrological monitoring and management**

- Ultrasound screening for malformations of a kidney and/or the urinary tract should be performed at diagnosis.
- Renal function (blood creatinine determinations) should be assessed in the event of renal malformations or hypertension, with subsequent monitoring, as determined by the specialist.
- Renal ultrasound with Doppler imaging of the renal arteries should be performed in cases of hypertension.
- Specialist nephrological care and monitoring (clinical, radiological) should be adapted to the renal abnormality.
- Preventive measures for urinary tract infections should be implemented for patients with abnormalities that tend to promote such infections. Appropriate treatment should be administered in cases of urinary tract infection.

### Management of metabolic abnormalities, overweight and bone tissue

**Management of metabolic abnormalities and overweight**

- Excess weight gain often begins in adolescence and can be associated with abnormalities in carbohydrate metabolism: insulin resistance, glucose intolerance, hyperinsulinism, or, more rarely, diabetes (type 1 or type 2) [80–84]. The recommendations regarding carbohydrate metabolism disorders were discussed in “Carbohydrate intolerance and diabetes” section.
- The risk of becoming overweight can be reduced by the early implementation of dietary measures, avoidance of an overly sedentary lifestyle and of the inclusion of physical activity in everyday activities [85].
- Dietary advice can be provided to patients who are not overweight (doctor and/or dietitian). In overweight patients, daily calorie intake should be evaluated, and hygiene and dietary measures should be established by the referring physician or a dietitian.
- Lipid panel [cholesterol (total, HDL, LDL), triglycerides] analyses should be performed between the ages of nine and 11 years in patients with risk factors (overweight, or family history of dyslipidemia), and then again between the ages of 17 and 21 years, and every three to four years thereafter, or annually in the event of cardiovascular risk factors.

**Orthopedic and osteopenia management**

- Patients with TS are at high risk of scoliosis and kyphosis during adolescence (10–20%), and of osteopenia and fractures due to estrogen deficiency, which can be remedied by appropriate long-term replacement therapy [86, 87].
- Progressive estrogen therapy, initiated at an appropriate pubertal age, optimizes peak bone mass acquisition and may therefore, at least to some extent, prevent osteopenia and the risk of subsequent fractures [88–90].

**Orthopedic monitoring and management**

- Screening for kyphosis or scoliosis should be performed annually, from the age of 8–10 years in particular, and every six months in patients on GH treatment. Spinal X rays should also be performed if necessary, and specialist advice obtained in the event of an abnormality on clinical examination.
- The doctor should check that calcium (1000 mg/day in the prepubertal period and 1200–1500 mg/day during puberty) and vitamin D intakes are appropriate for age during adolescence and adulthood.
- Screening for osteopenia should be performed by bone densitometry scan (DEXA), at the end of pediatric care or during the first adult consultation, and every five years thereafter, provided that there are no abnormalities (bone mineral density (BMD) adjusted for height).
- BMD check-up scans should be performed more frequently if BMD decreases, and possible contributory factors (poor compliance with HRT, alcohol and/or tobacco consumption, celiac disease) should also be investigated.
- The importance of continuing hormone replacement therapy at least until the physiological age of menopause (50 years), to prevent the risk of osteoporosis, should be explained to the patient.
- Specialist consultation would be appropriate in the event of osteoporosis, to ensure correct therapeutic management and follow-up.
- Regular physical activity should be encouraged.
- Foot deformities, which may become more pronounced with age (hallux valgus, flat feet) should be managed, and appropriate shoes should be worn, particularly if a short 4th metatarsal causes discomfort.

### Dermatological management

#### Management of lymphedema

Lymphedema is most common in the early postnatal period. It affects the hands and/or feet and generally regresses before the age of two years. However, it can recur at any age, particularly at the start of treatment with growth hormone or estrogen [91].

The use of slightly stretchable compression bandages (short-stretch bandages), put on by the girl herself or by with the help of her parents after training from a physiotherapist, during the night, and the wearing of elastic compression hose (socks) during the day may be recommended. Manual lymphatic drainage sessions may provide some comfort. Diuretics and vascular surgery should be avoided.

#### Management of cutaneous nevi

TS patients have a high frequency of nevi, but there does not seem to be a spontaneous increase in the risk of melanoma, even with GH treatment [92]. Moles should be checked at the time of TS diagnosis, and annually thereafter. Patients can be taught how to examine their own nevi to check for suspicious lesions. Nevi appear to be more pronounced during adolescence, and should be carefully monitored during this period.

#### Scar management

If there is a tendency for keloid scarring, this should be taken into account when considering indications for surgery in a patient with TS. Patients should also be warned that there is a risk of possible scarring problems following cosmetic piercing.

#### Management of psoriasis, alopecia

These manifestations are not particularly frequent and have been reported only occasionally. Treatments do not differ from those recommended for the general population.

### Dental management

Certain orofacial abnormalities of the ogival [high-arched] palate type or, much more rarely, cleft palate may be observed, as well as dental abnormalities (tooth agenesis, enamel abnormalities, hypoplasia, shorter tooth roots) and a high susceptibility to periodontitis [93, 94]. Premature tooth eruption is sometimes observed. Mandibular hypoplasia with retrognathia may lead to problems of malocclusion in some patients.

**Dental monitoring and management**

- Annual or biannual consultation with a dentist is recommended from early childhood, and throughout the period of growth, with education in oral-dental hygiene, the prescription of fluoride (tablets, toothpaste with a fluoride content appropriate for patient age), and the preventive sealing of pits and fissures, etc. A panoramic X ray can be performed after the age of seven years if the dentist deems it necessary.
- Consultation with an orthodontist may be organized, from the age of seven years (or earlier in the event of abnormalities), for orthodontic evaluation, if the dentist deems it necessary.
- Regular orthodontic follow-up may be required, depending on clinical manifestations (screening for temporomandibular joint disorder, etc.).
- Appropriate cautious orthodontic management (risk of tooth loosening) should be given, if necessary. A speech therapy assessment can also be performed, with rehabilitation if necessary.
- In adults, an annual consultation with a dentist is advised, with, in particular, careful monitoring of periodontal condition (these patients have a high risk of periodontitis).

### Ophthalmologic management

Ophthalmologic abnormalites are more frequent in TS patients than in the general population, but are not specific: refractive anomalies, strabismus, orbitopalpebral malformations, etc. Patients are at risk of amblyopia during the sensitive period of visual development (first decade of life) [95, 96].

**Ophthalmologic monitoring and management**

- Patients should be checked for suspicious signs or characteristic clinical signs at all ages:
  - Nystagmus/abnormal eye movements;
  - Behavior suggesting visual impairment;
  - Anatomical appearance of the eyes: white spot on or in the eye, eye too big or too small;
  - Pupils of different sizes;
  - Drooping eyelids;
  - Abnormal white, yellow, gray or red reflections in the eyes;
  - Eye deviation/pseudostrabismus/true strabismus.
- In the event of an early diagnosis of TS, systematic ophthalmologic examination should be performed at the age of 12–15 months (screening for amblyogenic factors = strabismus and/or refractive anomalies and/or organic abnormalities).
- Otherwise, systematic ophthalmologic examinations should be performed during the diagnostic assessment.
- The patients should undergo an ophthalmologic examination between the ages of three and four years, as in the general population. Examinations are required thereafter only in the presence of: 1) an ophthalmologic abnormality requiring specific and appropriate monitoring and management, the frequency of which is decided by the ophthalmologist; 2) the appearance of suspicious sensory or motor signs.

### Management of gastrointestinal and hepatic disorders

Eating difficulties (sucking and/or swallowing reflexes may be inefficient and/or associated with gastroesophageal reflux) are sometimes observed during the first few months or years of life. In addition, inflammatory digestive diseases (Crohn's disease, ulcerative colitis, chronic diarrhea) have been reported from adolescence (more frequent in cases of isochromosome Xq). The high risk of celiac disease was discussed in “Carbohydrate intolerance and diabetes” section.

Liver function abnormalities are common in TS patients and increase with age (30–50% at 35 years) [97–99]: cytolysis with ALT > AST, cholestasis, often with an increase in GGT levels, and sometimes both. The three most common types of liver damage are (in descending order of frequency of occurrence): (1) metabolic damage, such as steatosis and steatohepatitis; (2) vascular involvement with regenerative nodular hyperplasia. Cases of focal nodular hyperplasia (FNH), and benign liver tumors, have also been described; (3) biliary involvement of the primary sclerosing cholangitis type and autoimmune involvement of the primary biliary cholangitis (PBC) type. Abdominal Doppler ultrasound is important, to detect steatosis, nodules, fibrosis, and signs of portal hypertension (splenomegaly). By contrast, liver biopsy is an invasive procedure and is not systematically performed. FibroScan with elastography can be used to quantify fibrosis and steatosis, and may facilitate the selection of patients requiring biopsy or for whom it would be better to instigate more frequent follow-up. If this screening is positive for these abnormalities, serological tests for hepatitis B and C should be carried out, and the patients should be referred to a hepatologist for diagnosis and follow-up. Screening for complications, such as portal hypertension, which may require medical treatment with beta blockers, can be performed by gastrointestinal endoscopy. If cholestatic disease is isolated and ultrasound scan results are normal, treatment with ursodeoxycholic acid may be considered in the event of biliary involvement.

Gastrointestinal vascular abnormalities, such as telangiectasia and, more rarely, hemangioma, potentially leading to intermittent GI hemorrhage (melena, and even sometimes massive hemorrhage, for which surgery is often impossible due to the diffuse nature of the lesions), have been described. Estrogen seems to stabilize the bleeding, which would explain the improvement in symptoms with age, on replacement therapy.

**Hepato-gastroenterological monitoring and management**

- Possible gastroesophageal reflux should be treated.
- Given the risk of intermittent gastrointestinal hemorrhage, the doctor should check for signs suggestive of this condition during the medical interview.
- Careful monitoring, guided by the medical interview, and clinical examinations to screen for possible GI diseases should be performed.
- Liver function tests (AST, ALT, gamma-GT, PAL) should be performed annually, from the age of six years, with liver ultrasound scans if necessary (steatosis or hepatomegaly).
- In the event of abnormal liver function test results (values > N), viral serology tests should be performed, with follow-up testing six to 12 months later.
- In cases of laboratory test abnormalities, or the persistence of hepatosplenomegaly or clinical signs of portal hypertension, hepatobiliary ultrasound should be performed and the patient should be referred to a specialist clinic.
- In the presence of liver abnormalities, it is not necessary to stop hormone treatment with estrogens (unless the specialist advises otherwise). There is no contraindication to using the oral or transdermal route of administration in patients with such abnormalities. However, use of the transdermal route avoids first-pass hepatic metabolism.
- Weight loss is indicated to prevent or reduce steatosis.
- A specialist gastroenterology consultation with endoscopic exploration (capsule endoscopy of the small intestine or even upper GI endoscopies or ileocoloscopy, depending on the clinical profile) should be performed in the event of digestive tract bleeding and for evaluations of the impact of the condition (BP, clinical tolerance, CBC, ferritin).

### Fertility, contraception, sexuality

- Fertility: the patient should be offered a dedicated consultation with a specialist gynecologist, or an endocrinologist specializing in reproductive medicine, to provide information about the possibilities of preserving fertility and the possibilities for MAP, including, in particular, in vitro fertilization with egg donation, provided that there is no contraindication for pregnancy.
- Fertility preservation should be considered in children with preserved ovarian function (mosaicism), with the cryopreservation of ovarian tissue for prepubertal children. This technique remains experimental because no pregnancy after transplantation has been reported to date. Even now, this procedure is not recommended before the age of 10 years. Egg vitrification [flash-freezing] after ovarian stimulation can be used to preserve fertility in young women after puberty. However, very few data are currently available concerning the effectiveness of using frozen gametes.
- Contraception should be considered in patients with spontaneous cycles.
- Sexuality should be assessed during a specific dedicated consultation.

### Management of neurocognitive, psychological and psychosocial difficulties

Most patients have a normal intellectual capacity, but about 10% have mild-to-moderate learning impairment [100, 101]. The risk of mental retardation (usually very rare in TS) is higher in patients with a marker chromosome or a ring X chromosome, which does not contain the *XIST* gene. It is, therefore, linked to the absence of physiological inactivation of the chromosomal material present on this marker or ring chromosome. It should be remembered that one of the X chromosomes is almost entirely inactivated in individuals with an XX karyotype.

A particular neuropsychological profile, with difficulties in certain specific kinds of learning (calculation, mathematical problem solving, visiospatial orientation, attention, fine motor skills) is found in approximately 70% of cases [102, 103]. A hyperactive profile with attention deficit is sometimes encountered [104].

Behavioral features, such as isolation, anxiety, depression and some degree of immaturity are common in adolescence and adulthood [105]. Some patients may experience low self-esteem, with difficulties in their relationships and, particularly, their emotional life [106–108]*.* Infertility or certain complications (including ENT or heart problems) and a late onset of puberty appear to be determinant factors for quality of life and self-image [109–112]. Gender identity is clearly female.

Screening for neurocognitive, psychological and psychosocial difficulties and behavioral disorders should be performed at each assessment, and with particular care at the time of key transitions in school life.

**Neurocognitive, psychological and psychosocial monitoring and management**

- Parents should be informed about the possibility of a particular neuropsychological profile, with difficulties in certain specific types of learning, but should also be told that intellectual capacity is generally normal in TS patients.
- Information should be provided about the risk of cognitive disorders in the event of a ring X chromosome (absence of the *XIST* gene).
- Screening should be performed with psychometric tests for neurocognitive disorders (around the age of four or five years, earlier if there are suspicious signs, or during diagnostic investigation, regardless of the child’s age) as a function of clinical profile, with reassessment depending on symptoms.
- Appropriate management should be organized for patients with neurocognitive disorders.
- Psychological and quality-of-life evaluations should be carried out regularly, with appropriate management if necessary.
- Regular interviews, adapted to the patient's age, should be carried out with the patient, to assess and supplement knowledge about TS.
- It is important to check for socioprofessional integration problems. The assistance of a social worker or another type of personalized assistance can be recommended, if necessary.
- Information should be provided about the existence of patient associations.

If necessary, patients may be referred to an MDPH (a specialist authority for disabled people created in France by the law of February 11, 2005): these one-stop-shop establishments were set up to provide information, introductions and advice, to assess needs and to recommend a plan for personalized benefits and assistance, support and monitoring through an autonomy and rights committee for the disabled (CDAPH).

### Tumor risk

The incidence of breast cancer in TS patients is lower than that in the general population [49]*.* By contrast, TS patients have a higher risk of benign central nervous system tumors (meningiomas), cutaneous nevi that could potentially develop into melanoma, and colorectal cancer [113, 114].

The presence of Y chromosomal material is associated with a risk of gonadoblastoma (approximately 10%), which is mostly benign [115]. Malignancy is very rare.

#### Monitoring of tumor risk

- The possible presence of Y chromosome material should be checked in most patients, according to karyoptype (FISH, PCR, even CGH-array), and if such material is found, the possible presence of the GBY locus, located on the short arm of the Y, close to the centromere should be investigated.
- Prophylactic gonadectomy may be performed if Y material is found, and should be discussed at an MCM. Morphological monitoring by regular imaging (ultrasound, MRI) should be performed if a gonadectomy is refused. If girls have a satisfactory ovarian reserve at the end of puberty, they should systematically be offered a fertility preservation consultation before gonadectomy, once the girl’s consent has been obtained.
- Screening for breast and cervical cancer should be performed as in the general population: annual clinical breast monitoring from the age of 25 years and a mammogram every two years between the ages of 50 and 75 years; HPV tests and cervical smears should be performed in sexually active women between the ages of 25 and 65 years; HPV vaccination is recommended, in accordance with the national vaccination schedule.
- During the medical interview, patients should be asked about GI disorders, abdominal pain, and blood in the stools, to determine whether a diagnostic colonoscopy is necessary.
- Information should be provided about the moderate risk of colorectal cancer associated with TS. Colonoscopy may be recommended for screening purposes, every five years from the age of 45 years.
- Adults should undergo dermatological monitoring, preferably on an annual basis. Such screening for children should be discussed as a function of the significance of nevi.

#### TS and plastic surgery

- Information should be provided about the possibilities for cosmetic plastic surgery (pterygium colli, ear malformation, breast surgery, nevus removal, etc.).
- Information should be provided about the high risk of keloid scars (removal of nevi, piercing).

## Data Availability

Data sharing not applicable to this article as no datasets were generated or analyzed during the current study.

## Appendix 1: Clinical phenotype and potential associated disorders

Short stature and premature ovarian insufficiency affect all TS patients, but the other signs of TS may vary considerably between patients.

**Table:**

Phenotype, malformations and associated diseases/conditions	
**In the antenatal period: ultrasound signs**	
Thick neck, cystic hygroma, hydrops fetalis (anasarca)	
Aortic coarctation, left cardiac abnormalities	
Renal abnormalities	
Brachycephaly	
Hydramnios/oligoamnios	
Moderate intrauterine growth retardation	
**Newborn**	**Child/Adult**
Lymphedema, hands/feet	Short stature
Thick neck	Premature ovarian insufficiency
Intrauterine growth retardation	
**Eyes**	**Mouth**
Orbitopalpebral abnormalities (epicanthal folds, antimongoloid slant, ptosis), strabismus, refractive abnormalities	Micrognathia
	High-arched palate
	Dental abnormalities (agenesis, anomalous enamel structure)
**Ears**	**Neck**
Low-set	Low hairline
Malformations	Pterygium colli, short neck
Recurrent bouts of otitis	
Deafness	
**Skeleton**	**Heart–Aorta–Cardiovascular**
Cubitus valgus	Bicuspid aorta
Shortening of the fourth metacarpal bone	Aortic coarctation
	Aortic stenosis
Genu valgum	Aortic insufficiency
Madelung's deformity	Mitral prolapse
Scoliosis, kyphosis	Left heart hypoplasia
Bone maturation delay	Aortic dilation (± aneurysm, ± rupture)HBP
Low bone mineral density	
**Chest**	**Liver and GI**
Shield-shaped chest	Liver cytolysis
Widely spaced nipples	Celiac disease
Inverted nipples	Inflammatory GI diseases
**Endocrine glands**	**Kidneys–Bladder**
Autoimmune thyroiditis	Horseshoe kidney
Carbohydrate intolerance	Malrotation
Type 1 diabetes	Ectopic kidney
Type 2 diabetes	Renal agenesis
	Duplex kidney
	Hypodysplasia
	UTIs
**Skin and skin appendages**	**Neuropsychological aspects**
Multiple nevi	Learning difficulties
Lymphedema, hands/feet	Neurocognitive deficits
Vitiligo	Psychological immaturity
Alopecia	
Hypoplastic nails	

## Appendix 2: Initial investigations at the time of Turner Syndrome diagnosis.

Age at diagnosis.

**Table:**

	Neonatal	< 6 years	6–12 years	12–18 years	Adult
Detailed clinical examination, BP, screening for strabismus, kyphosis, scoliosis	X	X	X	X	X
Standard and Turner growth curves	X	X	X	X	
FSH ± LH ± estradiol ± AMH	X FSH, LH	X FSH, LH	X E2, FSH, LH, AMH	X E2, FSH, LH, AMH	X E2, FSH, LH, AMH
TSH, ± FT4		> 4 years	X	X	X
Anti-TPO antibodies		> 4 years	X	X	X
HbA1c ± fasting blood glucose			X (> 10 years)	X	X
± fasting insulin (HOMA)					
± OGTT		Before rhGH treatment if blood glucose and/or HbA1c levels are high	Before rhGH treatment if blood glucose and/or HbA1c levels are high	Before rhGH treatment if blood glucose and/or HbA1c levels are high	
AST, ALT, Gamma-GT, alkaline phosphatase			X > 6 years	X	X
IgA antitransglutaminases		X (> 2 years)	X	X	X As a function of symptoms
IgA total					
Blood creatinine microalbumin/creatinine in urine	If renal malformation	If renal malformation or HT	If renal malformation or HT	If renal malformation or HT	If renal malformation or HT
Total cholesterol/HDLc/LDLc/triglycerides				X	X
CBC			X	X	X
25(OH)D		X	X	X	X
Thrombophilia panel				Before estrogen/estrogen-progesterone treatments if there are thromboembolic risk factors (in first-degree relative)	Before estrogen/estrogen-progesterone treatments if there are thromboembolic risk factors (in first-degree relative)
Cardiology consultation	X	X	X	X	X
Echocardiogram	X	X	X	X	X
Electrocardiogram	X	X	X	X	X
Aortic and cardiac MRI		As advised by the cardiologist	As advised by the cardiologist	As advised by the cardiologist	As advised by the cardiologist
Renal ultrasound	X	X	X	X	X
Thyroid ultrasound		If dysthyroidism or nodule	If dysthyroidism or nodule	If dysthyroidism or nodule	If dysthyroidism or nodule
Bone age		X	X	X	
Pelvic ultrasound				After puberty	X
Bone densitometry				After puberty	X
ENT consultation and hearing assessment (adapted to age)		X	X	X	X
Ophthalmologic consultation	If suspicious signs	12–15 months and 3–4 yrs	If sensory or motor abnormality	If sensory or motor abnormality	If sensory or motor abnormality
Dental consultation ± panoramic X ray of dentition		If there is a clinical abnormality	X	X	X
Consultation with a dietitian		If overweight, carbohydrate intolerance, diabetes, dyslipidemia			
Psychological consultation ± psychometric tests	Parents, if necessary	X	X	X	X
Therapeutic education	Parents, if necessary	X	X	X	X

Karyotyping and screening for Y material should be redone if initially performed during the antenatal period.

## Appendix 3: Follow-up during pediatric (3.1) and adult (3.2) care.

### Pediatric care

**Table:**

	< 6 years	6–12 years	12–18 years
Pediatric endocrinology consultation	/1–3 years without GH	/1 year without GH	/6 months
	/6 months on rhGH	/6 months on rhGH	
Clinical examination	1x/year	/6 months on rhGH (1x/year without rhGH)	/6 months on rhGH (1x/year without rhGH)
Measurement of BP	1x/year	1x/year	1x/year
Screening for kyphosis, scoliosis (starting at 8–10 yrs++)	1x/year	1x/year	1x/year
	Every 6 months on GH	Every 6 months on rhGH	Every 6 months on rhGH
Standard and Turner growth curves	1x/year	At each visit	At each visit
Serum IGF-I determination	Start of GH treatment	Start of GH treatment	
	/12 months on rhGH treatment	/12 months on rhGH treatment	
Serum estradiol, FSH, LH, AMH determinations			Check before inducing puberty or 1x/year if puberty is spontaneous
Anti-TPO Ab	From the age of 4 years	1x/year	
TSH, ± FT4	From the age of 4 years	If treating hypo- or hyperthyroidism: TSH, FT4: every 3–12 months	
HbA1c		> 10 years: 1x/year HbA1C/3 months if diabetic	
± blood glucose levels			
± fasting insulinemia			
± OGTT	Before rhGH treatment if glycemia or HbA1c levels moderately high	OGTT before rhGH treatment if risk factors and if glycemia or HbA1c levels moderately high	
AST, ALT, gamma-GT		> 6 years:	
		6–10 years 1 × every 2 years	
		> 10 years: 1x/year	
IgA antitransglutaminases, total IgA	> 2 years: 1 × every 2 years	1 × every 2 years	1 × every 2 years
Total cholesterol/HDLc/LDLc/Triglycerides		> 10 years: 1 × every 3–4 years	1 × every 3–4 years
Creatininemia, Microalbumin/creatinine in urine	If renal malformation or HBP		
25(OH)D	/2–3 years	/2–3 years	/2–3 years
Consultation with (pediatric) cardiologist, with cardiac ultrasound	Depending on heart disease/condition	1x/year if cardiopathy or HBP	
		1 × every 5 years: systematically if no risk factors	
		Systematically before transition to adult care	
Aortic and cardiac MRI	As advised by the cardiologist	As advised by the cardiologist	
Thyroid ultrasound	If there is dysthyroidism, palpable nodule, and/or goiter		
ENT consultation and hearing evaluation	1x/year if recurrent bouts of otitis	As advised by ENT, with consultation at least once every 5 years provided there are no risk factors	
Ophthalmologic consultation	Systematic screening between 12 and 15 months and between 3 and 4 years (as in the general population)	Consultation only if necessary: suspicious signs, sensory or motor abnormality, follow-up for strabismus, follow-up of refractive abnormalities, follow-up for organic abnormality	
Dermatological consultation	As a function of symptoms		
Bone age	1 × every 2 to 3 years in case of rhGH treatment		
Bone densitometry		Check-up at end of the growth period or puberty	
Pelvic ultrasound		Check-up at end of the growth period or puberty	
Dental consultation	Annually, with adjustment according to clinical profile	Annually, with adjustment according to clinical profile	
Consultation with a dietitian	If overweight, carbohydrate intolerance, diabetes, dyslipidemia		
	Supplementation with calcium and vitamin D, if necessary		
Screening for behavioral problems	Every year, during the clinical evaluation		
Psychological consultation	Around the age of 4–5 years (before, if there are suspicious signs)	Every 5 years, with re-evaluation as a function of symptoms	
± Psychometric tests			
Therapeutic education	Every 1–2 years and as required		
Gynecology consultation		During puberty induction, then every 2–3 years	

### Adult care

**Table:**

	Adult period
Endocrinology ± gynecology consultation	1-2x/year
Clinical examination	1x/year
Measurement of BP	1x/year and more frequently if on antihypertensive medication
Follow-up karyotyping (± FISH)	If karyotyping was first performed more than 20 years ago
Anti-TPO antibodies	AB: every 1–2 years if anti-TPO Ab testing is negative
TSH	TSH, annual check if anti-TPO Ab are detected or if on levothyroxine treatment
HbA1c ± fasting blood glucose concentration	1x/year
± fasting insulinemia	HbA1C once every 3 months if diabetes
Serum estradiol, FSH, LH, AMH determinations	1x/year in patients with spontaneous cycles, after such cycles have stopped for at least two months or if on estrogen-progestagen contraceptive pill
AST, ALT, Gamma-GT, alkaline phosphatase	1x/year
Creatininemia, microalbumin/creatinine in urine	Frequency as advised by nephrologist (depending on the malformation)
	If high blood pressure
IgA antitransglutaminases, total IgA	1 × every 2–3 years or if there are suggestive symptoms
Cholesterol (Total/HDLc/LDLc/), triglycerides	1×/year
Cardiology consultation + echocardiogram	1 × every 5 years in the absence of cardiopathy and with normal annual BP measurements
	1 × every 6 months to 1×/year in cases of known cardiac disease and/or HT
Aortic and cardiac MRI	As advised by the cardiologist
Thyroid ultrasound	If dysthyroidism/palpated nodule/goiter
Pelvic ultrasound	Pre-conception
	Also helpful in the event of abnormal bleeding on THS or other gynecological symptoms
ENT consultation and hearing tests	Every 2–3 years, or more frequently if recommended by the ENT specialist
Ophthalmologic consultation	As a function of symptoms
Dermatological consultation	As a function of symptoms
Dental consultation	Annually (monitoring of periodontal condition)
Consultation with a dietitian	If overweight, carbohydrate intolerance, diabetes, dyslipidemia
Psychological consultation and social worker	If necessary
Therapeutic education	Every 1–5 years and otherwise as needed
Routine mammograms and cervical smears	Identical to recommendations for the general population
Colonoscopy	Recommended every five years from the age of 45 years
Bone densitometry	1 × every 5 years (more frequent if there is an abnormality)

## Appendix 5: Estrogen-progestogens for treating ovarian insufficiency.

**Table:**

Indication in the pubertal period	At what point?	Drugs
Estrogens	Around the age of 11 or 12 years	17-beta-estradiol: 0.2 mg/day
		(or 0.5 mg every other day) administration either oral or, preferably, transcutaneous: percutaneous delivery via gel or ¼ of a patch delivering 25 µg/24 h to be worn for 10–12 h at night
	After 12–24 months	17-beta-estradiol: 0.5 mg/d, administration oral or, preferably, transcutaneous: percutaneous delivery via gel or ½ a patch delivering 25 µg/24 h to be worn for 10–12 h at night
	When growth velocity < 2 cm/year: progressive or direct increase in dose	Increase doses of 17-beta-estradiol up to 2 mg/day orally or in gel form (1 to 1.5 mg/day) or by patch at 50–75 µg/24 h or 2 patches per week via the transcutaneous route
+ progesterone/progestogens (*)	Start when the estradiol dose reaches 0.8 to 1 mg/day or in case of metrorraghia	Minimum 12 days per month if discontinuous estrogen treatment and 14 days per month or every day if continuous treatment
	Or **combination of estrogen-progestogens** (**) either noncontraceptive or contraceptive	Orally: replacement dose

**(*) progestogens**

In combination with estradiol after a maximum of two to three years of estrogen-only treatment.

**Discontinuous sequential regimen (withdrawal bleeding at end of the month)**

17-beta-estradiol: 2 mg/day orally or 1 to 1.5 mg/day percutaneous 0.1% gel, (or 50–75 µg/day patch) transcutaneously from day 1 to day 25.

**Table:**

+	Micronized progesterone: 200 mg,	1 tab/day, from Day 14 to Day 25 (12 days)
or	dydrogesterone:	2 tabs/day, from Day 14 to Day 25 (12 days)

**Continuous sequential regimen (withdrawal bleeding at beginning of the month)**

17-beta-estradiol: 2 mg/day orally or percutaneous gel 1–1.5 mg/day or patch with 50–75 µg/24 h continuously.

**Table:**

+	Micronized progesterone: 200 mg,	1 tab/day for the last 14 days of the month
or	dydrogesterone:	2 tabs/day for the last 14 days of the month

**Continuous regimen (no withdrawal bleeding)**

17-beta-estradiol: 2 mg/day orally or percutaneous gel 1–1.5 mg/day or patch with 50–75 µg/24 h continuously.

**Table:**

+	Micronized progesterone:	1 tab/day continuously
or	100 mg, dydrogesterone:	1 tab/day continuously

**(**) noncontraceptive estrogen-progestogens**

Estradiol: 2 mg-dydrogesterone 10 mg (14 jours): 1 tab a day, every day

The lack of breast development after three months of estrogen treatment, or insufficient progression of breast development, are sufficient reason to recommend scaling up doses more quickly than in the suggested treatment regimen, or to switch to an oral route of administration, but only after ruling out non-compliance with the treatment regimen as a cause. It is best not to continue estrogen monotherapy for more than three years before introducing progestogens, unless the dose of estradiol prescribed remains low (≤ 0.5 mg/day). In this last case, the introduction of progestogens can be delayed and progestogen treatment may be initiated even if the dose of estradiol has not yet reached 2 mg/day.

If hypogonadism is observed after the onset of puberty, the initial estrogenization phase may be shortened to six months, or estrogen-progestogen treatment could even be prescribed from the outset, depending on breast development (for example 1 mg 17-beta-estradiol and 5 mg dydrogesterone for 14 days per month, for three to six months), as a function of clinical and uterine ultrasound assessments of estrogen impregnation.

Initial dose for inducing puberty: 1/10th the adult dose.

Introduction of progestogens after the first menstrual period or after 2–3 years of estrogen therapy.

Equivalences:

25 µg ethinylestradiol—2 mg 17 beta estradiol oral—1.5 mg percutaneous—50–75 µg transdermal.

## Appendix 6: Contacts

**CRMERCD Network- French Reference (CRMR) and Competence Centers (CCMR)**

**- Reference Centers**

**Assistance Publique–Hôpitaux de Paris, Université de Paris, and Sorbonne Université**

Prof. Carel Jean-Claude, Paris, France

Prof. Christin-Maitre Sophie, Paris, France

Prof. Léger Juliane, Paris, France

Prof. Netchine Irène, Paris, France

Prof. Polak Michel, Paris, France

Prof. Touraine Philippe, Paris, France

**- Competence expert Centers**

- Assistance Publique–Hôpitaux de Paris, Cochin site, Jérôme Bertherat (adult endocrinologist)
- University Hospital Center Amiens, Karine Braun (pediatric endocrinologist), Rachel Dessailloud (adult endocrinologist)
- University Hospital Center Angers, Régis Coutant (pediatric endocrinologist), Patrice Rodien (adult endocrinologist)
- University Hospital Center Besançon, Brigitte Mignot (pediatric endocrinologist), Franck Schillo (adult endocrinologist)
- University Hospital Center Brest, Karine Bourdet (pediatric endocrinologist), Véronique Kerlan (adult endocrinologist)
- University Hospital Center Caen, Virginie Ribault (pediatric endocrinologist), Yves Reznik (adult endocrinologist)
- University Hospital Center Clermont-Ferrand, Helene Malpuech-Rouffet (pediatric endocrinologist), Igor Tauveron (adult endocrinologist)
- University Hospital Center Dijon, Candace Bensignor (pediatric endocrinologist), Bruno Vergès (adult endocrinologist)
- University Hospital Center Grenoble, Clementine Dupuis (pediatric endocrinologist), Olivier Chabre (adult endocrinologist)
- University Hospital Center Limoges, Anne Lienhardt (pediatric endocrinologist), Marie-Pierre Teissier (adult endocrinologist)
- University Hospital Center Marseille, Rachel Reynaud (pediatric endocrinologist), Thierry Brue (adult endocrinologist)
- University Hospital Center Montpellier, Françoise Paris (pediatric endocrinologist), Eric Renard (adult endocrinologist)
- University Hospital Center Nancy, Emeline Renard (pediatric endocrinologist), Eva Feigerlova (adult endocrinologist)
- University Hospital Center Nantes, Sabine Baron (pediatric endocrinologist), Delphine Drui (adult endocrinologist)
- University Hospital Center Nice, Marie Hoflack (pediatric endocrinologist), Nicolas Chevalier (adult endocrinologist)
- University Hospital Center Reims, Pierre François Souchon (pediatric endocrinologist), Brigitte Delemer (adult endocrinologist)
- University Hospital Center Rennes, Sylvie Nivot-Adamiak (pediatric endocrinologist), Isabelle Guilhem (adult endocrinologist)
- University Hospital Center Réunion (Saint Pierre), Laure Houdon (pediatric endocrinologist)
- University Hospital Center Rouen, Mireille Castanet (pediatric endocrinologist), Hervé Lefebvre (adult endocrinologist)
- University Hospital Center Saint-Etienne, Odile Richard (pediatric endocrinologist), Natacha Germain (adult endocrinologist)
- University Hospital Center Strasbourg, Sylvie Soskin (pediatric endocrinologist), Nathalie Jeandidier (adult endocrinologist)
- University Hospital Center Toulouse, Maithé Tauber (pediatric endocrinologist), Philippe Caron (adult endocrinologist)
- University Hospital Center Tours, Myriam Bouillo (pediatric endocrinologist), Peggy Renoult-Pierre (adult endocrinologist)

**Patients’ Associations**

***AGAT Association***

*A.G.A.T (Association des Groupes Amitié Turner.* Association of Turner Syndrome Fellowship Groups*)*

*website:* http//www.agat-turner.org

*E-mail: association_agatts@yahoo.fr*

***Turner and You Association***

*website:* https://www.turneretvous.org/

*E-mail: contact@turneretvous.org*

***Grandir Association***

*website:* https://www.grandir.asso.fr/

**Other resources, General information**

- French Rare Endocrine Diseases network: http://www.firendo.fr:
- Portal for rare diseases and orphan drugs https://www.orpha.net/consor/cgibin/OC_Exp.php?lng=fr&Expert=881
- Rare Diseases Alliance: https://www.alliance-maladiesrares.org
- Rare Diseases Info Service: https://www.maladiesraresinfo.org/
- Rare Diseases Platform: https://www.plateforme-maladiesrares.org/
- Everyone in School: https://www.tousalecole.fr/
- French National Health Authority: https://www.has-sante.fr/
- French Society for Pediatric Endocrinology and Diabetes: https://sfedp.org/
- European Reference Network on Rare Endocrine Conditions (EndoERN): https://endo-ern.eu/

## Abbreviations

Ab: Antibody
ALP: Alkaline phosphatase
ALT: Alanine aminotransferase
AMH: Anti-Müllerian hormone
AP-HP: Assistance Publique-Hôpitaux de Paris [Paris Hospitals Public Assistance]
AST: Aspartate aminotransferase
BAV: Bicuspid aortic valve
BSA: Body surface area
BMD: Bone mineral density
BMI: Body mass index (W/H^2^)
BP: Blood pressure
CNS: Central nervous system
COA: Coarctation of the aorta
CPAP: Continuous positive airway pressure
CRMERCD: Centre de référence national des maladies endocriniennes rares de la croissance et du développement [Eng.: National Reference Center for Rare Growth and Developmental Endocrine Diseases]
DMO: Densité minérale osseuse [French]
EKG: Electrocardiogram
ENT: Ear-nose-throat specialist
FISH: Fluorescent in situ hybridization
NDCP: French National Diagnosis and Care Protocol
HAS: French National Health Authority, *Haute Autorité de Santé* [French]
FSH: Follicle-stimulating hormone
FT4: Free thyroxine
Gamma-GT: Gamma-glutamyltransferase
rhGH: Recombinant human growth hormone
HbA1c: Hemoglobin A1c
HDL: High-density lipoprotein
HOMA: Homeostasis model assessment of insulin resistance
HRT: Hormone replacement therapy
HT: Hypertension
IGF-I: Insulin-like growth factor-I
BMI: Body mass index (*P/T*^2^)\
IUGR: Intrauterine growth retardation
IVF: In vitro fertilization
LDL: Low-density lipoprotein
LFT: Liver function testing/panel
LH: Luteinizing hormone
ALD: Affection de Longue durée. Long-term condition justifying 100% coverage by the French social security system
MAR: Medically assisted reproduction
MDPH: Maison départementale pour les personnes handicapées [French]
MCM: Multidisciplinary coordination meeting
NAAHE: National Agency for Health Accreditation and Evaluation
OGTT: Oral glucose tolerance test
PCR: Polymerase chain reaction
PNDS: Protocole national de diagnostic et de soins [French]
RCP: Réunion de Concertation Pluridisciplinaire [French]
RCDP: Regional centers for disabled people
SDS: Standard deviation score
TPO: Thyroperoxidase
TS: Turner Syndrome
TSH: Thyroid-stimulating hormone

## References

[1] Gravholt CH, Andersen NH, Conway GS, Dekkers OM, Geffner ME, Klein KO (2017). Clinical practice guidelines for the care of girls and women with Turner syndrome: proceedings from the 2016 Cincinnati International Turner Syndrome Meeting. Eur J Endocrinol.

[2] Massa G, Verlinde F, De Schepper J, Thomas M, Bourguignon JP, Craen M (2005). Trends in age at diagnosis of Turner syndrome. Arch Dis Child.

[3] Bernard V, Donadille B, Le Poulennec T, Nedelcu M, Martinerie L, Christin-Maitre S (2019). Management of endocrine disease: transition of care for young adult patients with Turner syndrome. Eur J Endocrinol.

[4] Fiot E, Zenaty D, Boizeau P, Haignere J, Santos SD, Leger J (2016). X-chromosome gene dosage as a determinant of impaired pre and postnatal growth and adult height in Turner syndrome. Eur J Endocrinol.

[5] Lin AE, Prakash SK, Andersen NH, Viuff MH, Levitsky LL, Rivera-Davila M (2019). Recognition and management of adults with Turner syndrome: from the transition of adolescence through the senior years. Am J Med Genet A.

[6] Li P, Cheng F, Xiu L (2018). Height outcome of the recombinant human growth hormone treatment in Turner syndrome: a meta-analysis. Endocr Connect.

[7] Soriano-Guillen L, Coste J, Ecosse E, Leger J, Tauber M, Cabrol S (2005). Adult height and pubertal growth in Turner syndrome after treatment with recombinant growth hormone. J Clin Endocrinol Metab.

[8] Ranke MB, Lindberg A, Ferrandez Longas A, Darendeliler F, Albertsson-Wikland K, Dunger D (2007). Major determinants of height development in Turner syndrome (TS) patients treated with GH: analysis of 987 patients from KIGS. Pediatr Res.

[9] Gault EJ, Cole TJ, Casey S, Hindmarsh PC, Betts P, Dunger DB (2021). Effect of oxandrolone and timing of pubertal induction on final height in Turner syndrome: final analysis of the UK randomised placebo-controlled trial. Arch Dis Child.

[10] Lanes R, Lindberg A, Carlsson M, Chrysis D, Aydin F, Camacho-Hubner C (2019). Near adult height in girls with turner syndrome treated with growth hormone following either induced or spontaneous puberty. J Pediatr.

[11] Quigley CA, Fechner PY, Geffner ME, Eugster EA, Ross JL, Habiby RL (2021). Prevention of growth failure in turner syndrome: long-term results of early growth hormone treatment in the "Toddler Turner" cohort. Horm Res Paediatr.

[12] Child CJ, Zimmermann AG, Chrousos GP, Cummings E, Deal CL, Hasegawa T (2019). Safety outcomes during pediatric GH therapy: final results from the prospective GeNeSIS observational program. J Clin Endocrinol Metab.

[13] Stochholm K, Juul S, Juel K, Naeraa RW, Gravholt CH (2006). Prevalence, incidence, diagnostic delay, and mortality in Turner syndrome. J Clin Endocrinol Metab.

[14] Bell J, Parker KL, Swinford RD, Hoffman AR, Maneatis T, Lippe B (2010). Long-term safety of recombinant human growth hormone in children. J Clin Endocrinol Metab.

[15] Davenport ML, Roush J, Liu C, Zagar AJ, Eugster E, Travers S (2010). Growth hormone treatment does not affect incidences of middle ear disease or hearing loss in infants and toddlers with Turner syndrome. Horm Res Paediatr.

[16] Swerdlow AJ, Cooke R, Beckers D, Butler G, Carel JC, Cianfarani S (2019). Risk of meningioma in European patients treated with growth hormone in childhood: results from the SAGhE cohort. J Clin Endocrinol Metab.

[17] Swerdlow AJ, Cooke R, Beckers D, Borgstrom B, Butler G, Carel JC (2017). Cancer risks in patients treated with growth hormone in childhood: the SAGhE European Cohort Study. J Clin Endocrinol Metab.

[18] Bernard V, Donadille B, Zenaty D, Courtillot C, Salenave S, Brac-de-la-Perriere A (2016). Spontaneous fertility and pregnancy outcomes amongst 480 women with Turner syndrome. Hum Reprod.

[19] Donaldson M, Kristrom B, Ankarberg-Lindgren C, Verlinde S, van Alfen van der Velden J, Gawlik A (2019). Optimal pubertal induction in girls with turner syndrome using either oral or transdermal estradiol: a proposed modern strategy. Horm Res Paediatr..

[20] Klein KO, Rosenfield RL, Santen RJ, Gawlik AM, Backeljauw PF, Gravholt CH (2018). Estrogen replacement in turner syndrome: literature review and practical considerations. J Clin Endocrinol Metab.

[21] Zaiem F, Alahdab F, Al Nofal A, Murad MH, Javed A (2017). Oral versus transdermal Estrogen in Turner syndrome: a systematic review and meta-analysis. Endocr Pract.

[22] Torres-Santiago L, Mericq V, Taboada M, Unanue N, Klein KO, Singh R (2013). Metabolic effects of oral versus transdermal 17beta-estradiol (E(2)): a randomized clinical trial in girls with Turner syndrome. J Clin Endocrinol Metab.

[23] European Society for Human R, Embryology Guideline Group on POI, Webber L, Davies M, Anderson R, Bartlett J, et al. ESHRE Guideline: management of women with premature ovarian insufficiency. Hum Reprod. 2016;31(5):926–37 10.1093/humrep/dew027 27008889

[24] Bondy CA, Turner Syndrome Study G. Care of girls and women with Turner syndrome: a guideline of the Turner Syndrome Study Group. J Clin Endocrinol Metab. 2007;92(1):10–25. 10.1210/jc.2006-1374 17047017

[25] Nordenstrom A, Ahmed SF, van den Akker E, Blair J, Bonomi M, Brachet C (2022). Pubertal induction and transition to adult sex hormone replacement in patients with congenital pituitary or gonadal reproductive hormone deficiency: an Endo-ERN clinical practice guideline. Eur J Endocrinol.

[26] Folsom LJ, Slaven JE, Nabhan ZM, Eugster EA (2017). Characterization of spontaneous and induced puberty in girls with turner syndrome. Endocr Pract.

[27] Hage M, Plesa O, Lemaire I, Raffin Sanson ML (2022). Estrogen and progesterone therapy and meningiomas. Endocrinology.

[28] Sullivan SD, Sarrel PM, Nelson LM (2016). Hormone replacement therapy in young women with primary ovarian insufficiency and early menopause. Fertil Steril.

[29] Cameron-Pimblett A, Davies MC, Burt E, Talaulikar VS, La Rosa C, King TFJ (2019). Effects of Estrogen therapies on outcomes in turner syndrome: assessment of induction of puberty and adult Estrogen use. J Clin Endocrinol Metab.

[30] Christ JP, Gunning MN, Palla G, Eijkemans MJC, Lambalk CB, Laven JSE (2018). Estrogen deprivation and cardiovascular disease risk in primary ovarian insufficiency. Fertil Steril..

[31] Viuff MH, Berglund A, Juul S, Andersen NH, Stochholm K, Gravholt CH (2020). Sex hormone replacement therapy in turner syndrome: impact on morbidity and mortality. J Clin Endocrinol Metab..

[32] Roulot D, Degott C, Chazouilleres O, Oberti F, Cales P, Carbonell N (2004). Vascular involvement of the liver in Turner's syndrome. Hepatology.

[33] Cadoret F, Parinaud J, Bettiol C, Pienkowski C, Letur H, Ohl J (2018). Pregnancy outcome in Turner syndrome: a French multi-center study after the 2009 guidelines. Eur J Obstet Gynecol Reprod Biol.

[34] Chevalier N, Letur H, Lelannou D, Ohl J, Cornet D, Chalas-Boissonnas C (2011). Materno-fetal cardiovascular complications in Turner syndrome after oocyte donation: insufficient prepregnancy screening and pregnancy follow-up are associated with poor outcome. J Clin Endocrinol Metab.

[35] Donadille B, Bernard V, Christin-Maitre S (2019). How can we make pregnancy safe for women with Turner syndrome?. Am J Med Genet C Semin Med Genet.

[36] Chalas Boissonnas C, Davy C, Marszalek A, Duranteau L, de Ziegler D, Wolf JP (2011). Cardiovascular findings in women suffering from Turner syndrome requesting oocyte donation. Hum Reprod.

[37] Bakalov VK, Gutin L, Cheng CM, Zhou J, Sheth P, Shah K (2012). Autoimmune disorders in women with turner syndrome and women with karyotypically normal primary ovarian insufficiency. J Autoimmun.

[38] Mortensen KH, Cleemann L, Hjerrild BE, Nexo E, Locht H, Jeppesen EM (2009). Increased prevalence of autoimmunity in Turner syndrome–influence of age. Clin Exp Immunol.

[39] Marild K, Stordal K, Hagman A, Ludvigsson JF (2016). Turner syndrome and celiac disease: a case-control study. Pediatrics.

[40] Wegiel M, Antosz A, Gieburowska J, Szeliga K, Hankus M, Grzybowska-Chlebowczyk U (2019). Autoimmunity predisposition in girls with turner syndrome. Front Endocrinol (Lausanne).

[41] Wasniewska M, Salerno M, Corrias A, Mazzanti L, Matarazzo P, Corica D (2016). The evolution of thyroid function after presenting with hashimoto thyroiditis is different between initially Euthyroid girls with and those without turner syndrome. Horm Res Paediatr.

[42] Ibarra-Gasparini D, Altieri P, Scarano E, Perri A, Morselli-Labate AM, Pagotto U (2018). New insights on diabetes in Turner syndrome: results from an observational study in adulthood. Endocrine.

[43] Mortensen KH, Andersen NH, Gravholt CH (2012). Cardiovascular phenotype in Turner syndrome–integrating cardiology, genetics, and endocrinology. Endocr Rev.

[44] Corbitt H, Gutierrez J, Silberbach M, Maslen CL (2019). The genetic basis of Turner syndrome aortopathy. Am J Med Genet C Semin Med Genet.

[45] Bondy C, Bakalov VK, Cheng C, Olivieri L, Rosing DR, Arai AE (2013). Bicuspid aortic valve and aortic coarctation are linked to deletion of the X chromosome short arm in Turner syndrome. J Med Genet.

[46] Kim HK, Gottliebson W, Hor K, Backeljauw P, Gutmark-Little I, Salisbury SR (2011). Cardiovascular anomalies in Turner syndrome: spectrum, prevalence, and cardiac MRI findings in a pediatric and young adult population. AJR Am J Roentgenol.

[47] Mortensen KH, Hjerrild BE, Andersen NH, Sorensen KE, Horlyck A, Pedersen EM (2010). Abnormalities of the major intrathoracic arteries in Turner syndrome as revealed by magnetic resonance imaging. Cardiol Young.

[48] Subramaniam DR, Stoddard WA, Mortensen KH, Ringgaard S, Trolle C, Gravholt CH (2017). Continuous measurement of aortic dimensions in Turner syndrome: a cardiovascular magnetic resonance study. J Cardiovasc Magn Reson.

[49] Schoemaker MJ, Swerdlow AJ, Higgins CD, Wright AF, Jacobs PA, United Kingdom Clinical Cytogenetics G. Mortality in women with turner syndrome in Great Britain: a national cohort study. J Clin Endocrinol Metab. 2008;93(12):4735–42. 10.1210/jc.2008-1049 18812477

[50] Fuchs MM, Attenhofer Jost C, Babovic-Vuksanovic D, Connolly HM, Egbe A (2019). Long-term outcomes in patients with turner syndrome: a 68-year follow-up. J Am Heart Assoc.

[51] Thunstrom S, Krantz E, Thunstrom E, Hanson C, Bryman I, Landin-Wilhelmsen K (2019). Incidence of aortic dissection in turner syndrome. Circulation.

[52] Duijnhouwer AL, Bons LR, Timmers H, van Kimmenade RRL, Snoeren M, Timmermans J (2019). Aortic dilatation and outcome in women with Turner syndrome. Heart.

[53] Donadille B, Tuffet S, Cholet C, Nedelcu M, Bourcigaux N, Iserin L (2020). Prevalence and progression of aortic dilatation in adult patients with Turner syndrome: a cohort study. Eur J Endocrinol.

[54] Mortensen KH, Wen J, Erlandsen M, Trolle C, Ringgaard S, Gutmark EJ (2019). Aortic growth rates are not increased in Turner syndrome-a prospective CMR study. Eur Heart J Cardiovasc Imaging.

[55] Silberbach M, Roos-Hesselink JW, Andersen NH, Braverman AC, Brown N, Collins RT (2018). Cardiovascular health in turner syndrome: a scientific statement from the American Heart Association. Circ Genom Precis Med.

[56] Viuff MH, Trolle C, Wen J, Jensen JM, Norgaard BL, Gutmark EJ (2016). Coronary artery anomalies in Turner syndrome. J Cardiovasc Comput Tomogr.

[57] Wen J, Trolle C, Viuff MH, Ringgaard S, Laugesen E, Gutmark EJ (2018). Impaired aortic distensibility and elevated central blood pressure in Turner Syndrome: a cardiovascular magnetic resonance study. J Cardiovasc Magn Reson.

[58] De Groote K, Demulier L, De Backer J, De Wolf D, De Schepper J, T'Sjoen G (2015). Arterial hypertension in Turner syndrome: a review of the literature and a practical approach for diagnosis and treatment. J Hypertens.

[59] Sandahl K, Wen J, Erlandsen M, Andersen NH, Gravholt CH (2020). Natural history of hypertension in Turner syndrome during a 12-year pragmatic interventional study. Hypertension.

[60] Los E, Quezada E, Chen Z, Lapidus J, Silberbach M (2016). Pilot study of blood pressure in girls with turner syndrome: an awareness gap, clinical associations, and new hypotheses. Hypertension.

[61] Brun S, Berglund A, Mortensen KH, Hjerrild BE, Hansen KW, Andersen NH (2019). Blood pressure, sympathovagal tone, exercise capacity and metabolic status are linked in Turner syndrome. Clin Endocrinol (Oxf).

[62] Noordman ID, Duijnhouwer AL, Coert M, Bos M, Kempers M, Timmers H (2020). No QTc Prolongation in Girls and Women with Turner Syndrome. J Clin Endocrinol Metab..

[63] Quezada E, Lapidus J, Shaughnessy R, Chen Z, Silberbach M (2015). Aortic dimensions in Turner syndrome. Am J Med Genet A.

[64] Roman MJ, Devereux RB, Kramer-Fox R, O'Loughlin J (1989). Two-dimensional echocardiographic aortic root dimensions in normal children and adults. Am J Cardiol.

[65] Pater CM, Gutmark-Little I, Tretter JT, Martin LJ, Backeljauw P, Brown NM (2021). Clinical characteristics and rate of dilatation in Turner syndrome patients treated for aortic dilatation. Am J Med Genet A.

[66] Cabanes L, Chalas C, Christin-Maitre S, Donadille B, Felten ML, Gaxotte V (2010). Turner syndrome and pregnancy: clinical practice. Recommendations for the management of patients with Turner syndrome before and during pregnancy. Eur J Obstet Gynecol Reprod Biol..

[67] Grewal J, Valente AM, Egbe AC, Wu FM, Krieger EV, Sybert VP (2021). Cardiovascular outcomes of pregnancy in Turner syndrome. Heart.

[68] Calanchini M, Aye CYL, Orchard E, Baker K, Child T, Fabbri A (2020). Fertility issues and pregnancy outcomes in Turner syndrome. Fertil Steril.

[69] Fiot E, Zenaty D, Boizeau P, Haignere J, Dos Santos S, Leger J (2019). X chromosome gene dosage as a determinant of congenital malformations and of age-related comorbidity risk in patients with Turner syndrome, from childhood to early adulthood. Eur J Endocrinol.

[70] King KA, Makishima T, Zalewski CK, Bakalov VK, Griffith AJ, Bondy CA (2007). Analysis of auditory phenotype and karyotype in 200 females with Turner syndrome. Ear Hear.

[71] Bonnard A, Hederstierna C, Bark R, Hultcrantz M (2017). Audiometric features in young adults with Turner syndrome. Int J Audiol.

[72] Alvarez-Nava F, Racines-Orbe M, Witt J, Guarderas J, Vicuna Y, Estevez M (2020). Metabolic syndrome as a risk factor for sensorineural hearing loss in adult patients with turner syndrome. Appl Clin Genet.

[73] Bonnard A, Bark R, Hederstierna C (2019). Clinical update on sensorineural hearing loss in Turner syndrome and the X-chromosome. Am J Med Genet C Semin Med Genet.

[74] Hamberis AO, Mehta CH, Dornhoffer JR, Meyer TA (2020). Characteristics and progression of hearing loss in children with turner's syndrome. Laryngoscope.

[75] Lippe B, Geffner ME, Dietrich RB, Boechat MI, Kangarloo H (1988). Renal malformations in patients with Turner syndrome: imaging in 141 patients. Pediatrics.

[76] Bilge I, Kayserili H, Emre S, Nayir A, Sirin A, Tukel T (2000). Frequency of renal malformations in Turner syndrome: analysis of 82 Turkish children. Pediatr Nephrol.

[77] Sybert VP, McCauley E (2004). Turner's syndrome. N Engl J Med.

[78] Je BK, Kim HK, Horn PS (2015). Incidence and spectrum of renal complications and extrarenal diseases and syndromes in 380 children and young adults with horseshoe kidney. AJR Am J Roentgenol.

[79] Ogawa T, Takizawa F, Mukoyama Y, Ogawa A, Ito J (2021). Renal morphology and function from childhood to adulthood in Turner syndrome. Clin Exp Nephrol.

[80] O'Gorman CS, Syme C, Lang J, Bradley TJ, Wells GD, Hamilton JK (2013). An evaluation of early cardiometabolic risk factors in children and adolescents with Turner syndrome. Clin Endocrinol (Oxf).

[81] Davis SM, Geffner ME (2019). Cardiometabolic health in Turner syndrome. Am J Med Genet C Semin Med Genet.

[82] Sun L, Wang Y, Zhou T, Zhao X, Wang Y, Wang G (2019). Glucose metabolism in turner syndrome. Front Endocrinol (Lausanne).

[83] Lebenthal Y, Levy S, Sofrin-Drucker E, Nagelberg N, Weintrob N, Shalitin S (2018). The natural history of metabolic comorbidities in turner syndrome from childhood to early adulthood: comparison between 45, X monosomy and other karyotypes. Front Endocrinol (Lausanne).

[84] Cameron-Pimblett A, La Rosa C, King TFJ, Davies MC, Conway GS (2017). The Turner syndrome life course project: karyotype-phenotype analyses across the lifespan. Clin Endocrinol (Oxf).

[85] Thompson T, Zieba B, Howell S, Karakash W, Davis S (2020). A mixed methods study of physical activity and quality of life in adolescents with Turner syndrome. Am J Med Genet A.

[86] Gravholt CH, Vestergaard P, Hermann AP, Mosekilde L, Brixen K, Christiansen JS (2003). Increased fracture rates in Turner's syndrome: a nationwide questionnaire survey. Clin Endocrinol (Oxf).

[87] Nour MA, Burt LA, Perry RJ, Stephure DK, Hanley DA, Boyd SK (2016). Impact of growth hormone on adult bone quality in turner syndrome: a HR-pQCT study. Calcif Tissue Int.

[88] Shi K, Liu L, He YJ, Li D, Yuan LX, Lash GE (2016). Body composition and bone mineral status in patients with Turner syndrome. Sci Rep.

[89] Holroyd CR, Davies JH, Taylor P, Jameson K, Rivett C, Cooper C (2010). Reduced cortical bone density with normal trabecular bone density in girls with Turner syndrome. Osteoporos Int.

[90] Soucek O, Schonau E, Lebl J, Willnecker J, Hlavka Z, Sumnik Z (2018). A 6-year follow-up of fracture incidence and volumetric bone mineral density development in girls with turner syndrome. J Clin Endocrinol Metab.

[91] Atton G, Gordon K, Brice G, Keeley V, Riches K, Ostergaard P (2015). The lymphatic phenotype in Turner syndrome: an evaluation of nineteen patients and literature review. Eur J Hum Genet.

[92] Brazzelli V, Larizza D, Martinetti M, Martinoli S, Calcaterra V, De Silvestri A (2004). Halo nevus, rather than vitiligo, is a typical dermatologic finding of turner's syndrome: clinical, genetic, and immunogenetic study in 72 patients. J Am Acad Dermatol.

[93] Cazzolla AP, Lo Muzio L, Di Fede O, Lacarbonara V, Colaprico A, Testa NF (2018). Orthopedic-orthodontic treatment of the patient with Turner's syndrome: Review of the literature and case report. Spec Care Dentist.

[94] Ahiko N, Baba Y, Tsuji M, Horikawa R, Moriyama K (2019). Investigation of maxillofacial morphology and oral characteristics with Turner syndrome and early mixed dentition. Congenit Anom (Kyoto).

[95] Denniston AK, Butler L (2004). Ophthalmic features of Turner's syndrome. Eye (Lond).

[96] Wikiera B, Mulak M, Koltowska-Haggstrom M, Noczynska A (2015). The presence of eye defects in patients with Turner syndrome is irrespective of their karyotype. Clin Endocrinol (Oxf).

[97] Roulot D (2013). Liver involvement in Turner syndrome. Liver Int.

[98] Lee MC, Conway GS (2013). Liver dysfunction in Turner syndrome and its relationship to exogenous oestrogen. Eur J Gastroenterol Hepatol.

[99] Calanchini M, Moolla A, Tomlinson JW, Cobbold JF, Grossman A, Fabbri A (2018). Liver biochemical abnormalities in Turner syndrome: a comprehensive characterization of an adult population. Clin Endocrinol (Oxf).

[100] Hutaff-Lee C, Bennett E, Howell S, Tartaglia N (2019). Clinical developmental, neuropsychological, and social-emotional features of Turner syndrome. Am J Med Genet C Semin Med Genet.

[101] Bispo AV, Dos Santos LO, Buregio-Frota P, Galdino MB, Duarte AR, Leal GF (2013). Effect of chromosome constitution variations on the expression of Turner phenotype. Genet Mol Res.

[102] Temple CM, Shephard EE (2012). Exceptional lexical skills but executive language deficits in school starters and young adults with Turners syndrome: implications for X chromosome effects on brain function. Brain Lang.

[103] Anaki D, Zadikov-Mor T, Gepstein V, Hochberg Z (2018). Normal performance in non-visual social cognition tasks in women with turner syndrome. Front Endocrinol (Lausanne).

[104] Green T, Bade Shrestha S, Chromik LC, Rutledge K, Pennington BF, Hong DS (2015). Elucidating X chromosome influences on attention deficit hyperactivity disorder and executive function. J Psychiatr Res.

[105] Morris LA, Tishelman AC, Kremen J, Ross RA (2020). Depression in turner syndrome: a systematic review. Arch Sex Behav.

[106] Schmidt PJ, Cardoso GM, Ross JL, Haq N, Rubinow DR, Bondy CA (2006). Shyness, social anxiety, and impaired self-esteem in Turner syndrome and premature ovarian failure. JAMA.

[107] Hong DS, Dunkin B, Reiss AL (2011). Psychosocial functioning and social cognitive processing in girls with Turner syndrome. J Dev Behav Pediatr.

[108] Stochholm K, Hjerrild B, Mortensen KH, Juul S, Frydenberg M, Gravholt CH (2012). Socioeconomic parameters and mortality in Turner syndrome. Eur J Endocrinol.

[109] Carel JC, Elie C, Ecosse E, Tauber M, Leger J, Cabrol S (2006). Self-esteem and social adjustment in young women with Turner syndrome–influence of pubertal management and sexuality: population-based cohort study. J Clin Endocrinol Metab.

[110] Carel JC, Ecosse E, Bastie-Sigeac I, Cabrol S, Tauber M, Leger J (2005). Quality of life determinants in young women with turner's syndrome after growth hormone treatment: results of the StaTur population-based cohort study. J Clin Endocrinol Metab.

[111] Krantz E, Landin-Wilhelmsen K, Trimpou P, Bryman I, Wide U (2019). Health-related quality of life in turner syndrome and the influence of growth hormone therapy: a 20-year follow-up. J Clin Endocrinol Metab.

[112] van den Hoven AT, Bons LR, Dykgraaf RHM, Dessens AB, Pastoor H, de Graaff LCG (2020). A value-based healthcare approach: health-related quality of life and psychosocial functioning in women with Turner syndrome. Clin Endocrinol (Oxf).

[113] Larizza D, Albanesi M, De Silvestri A, Accordino G, Brazzelli V, Maffe GC (2016). Neoplasia in Turner syndrome. The importance of clinical and screening practices during follow-up. Eur J Med Genet..

[114] Viuff MH, Stochholm K, Lin A, Berglund A, Juul S, Gravholt CH (2021). Cancer occurrence in Turner syndrome and the effect of sex hormone substitution therapy. Eur J Endocrinol.

[115] Kwon A, Hyun SE, Jung MK, Chae HW, Lee WJ, Kim TH (2017). Risk of gonadoblastoma development in patients with turner syndrome with cryptic Y chromosome material. Horm Cancer.

